# Supplementary material for: How predictability affects habituation to novelty
Source: PLoS One. 2021 Jun 1;16(6):e0237278. doi: 10.1371/journal.pone.0237278 (PMC8168884; doi:10.1371/journal.pone.0237278)
Supplement: S1 File — (ZIP) [file pone.0237278.s001.zip › supplementary material/sup_math_deriv.docx]

Assume a Bayesian posterior given n times stimuli *s*(*n*) follows Gaussian distribution.

where and are variation of prior and variation of likelihood, respectively. is learning rate.

When n-1 th posterior is used as n th prior, the information gain given n th stimulus is KL-divergance between the prior and the posterior. The KL-divergence comprises the three integral terms.

Each integral term is derived as follows:

, where

The information gain is derived as a quadratic function of prediction error .

, where
