# Supplementary figures and images for: How predictability affects habituation to novelty
Source: PLoS One. 2021 Jun 1;16(6):e0237278. doi: 10.1371/journal.pone.0237278 (PMC8168884; doi:10.1371/journal.pone.0237278)

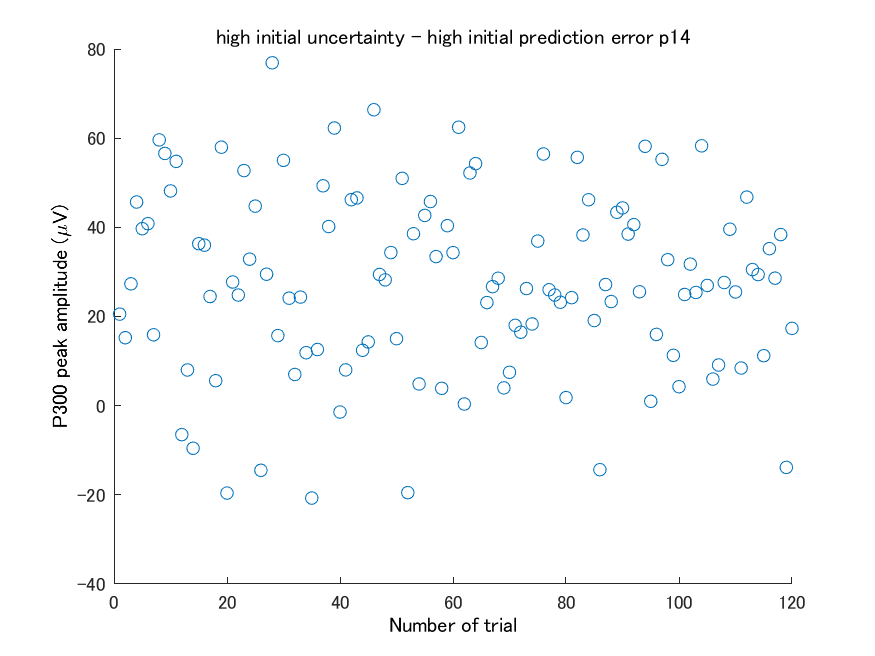

Supplement: S1 File — (ZIP) [file pone.0237278.s001.zip › supplementary material/high initial uncertainty_high initial prediction error_p014.tif]

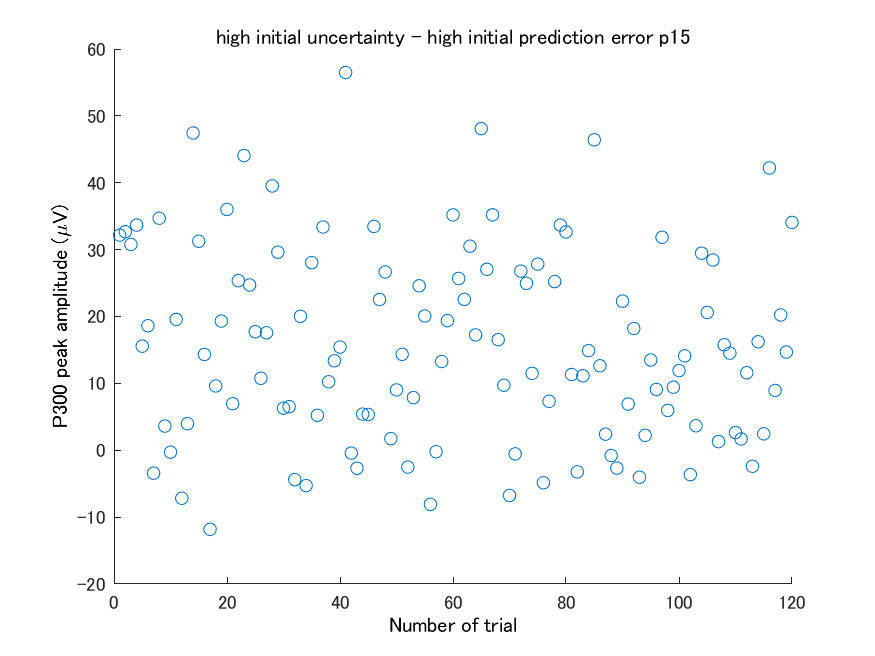

Supplement: S1 File — (ZIP) [file pone.0237278.s001.zip › supplementary material/high initial uncertainty_high initial prediction error_p015.tif]

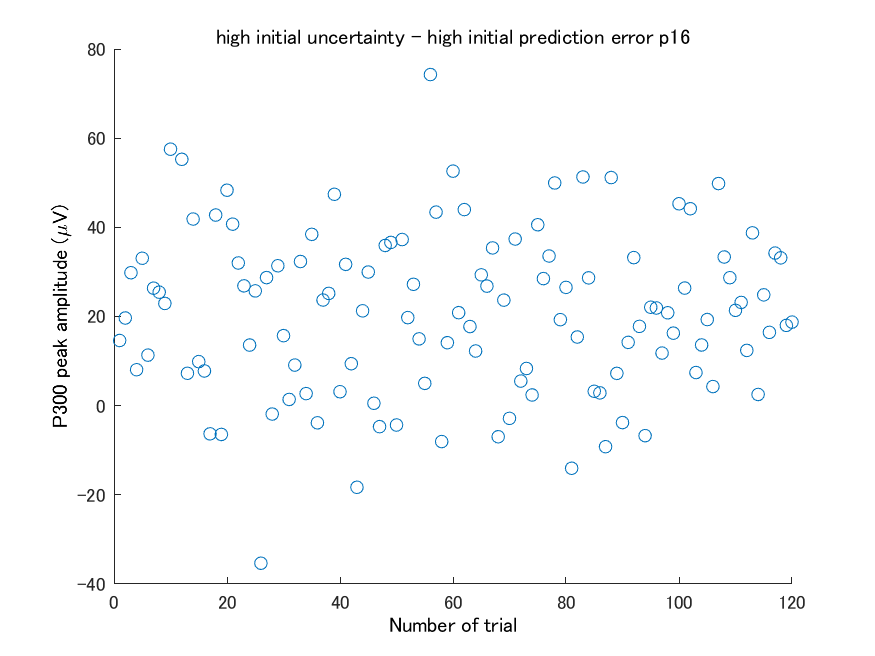

Supplement: S1 File — (ZIP) [file pone.0237278.s001.zip › supplementary material/high initial uncertainty_high initial prediction error_p016.tif]

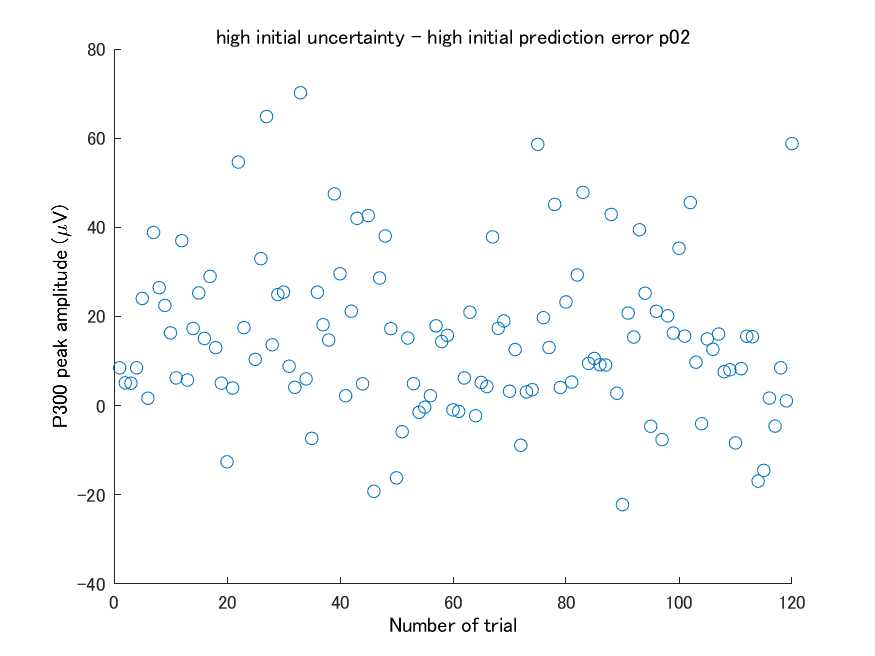

Supplement: S1 File — (ZIP) [file pone.0237278.s001.zip › supplementary material/high initial uncertainty_high initial prediction error_p02.tif]

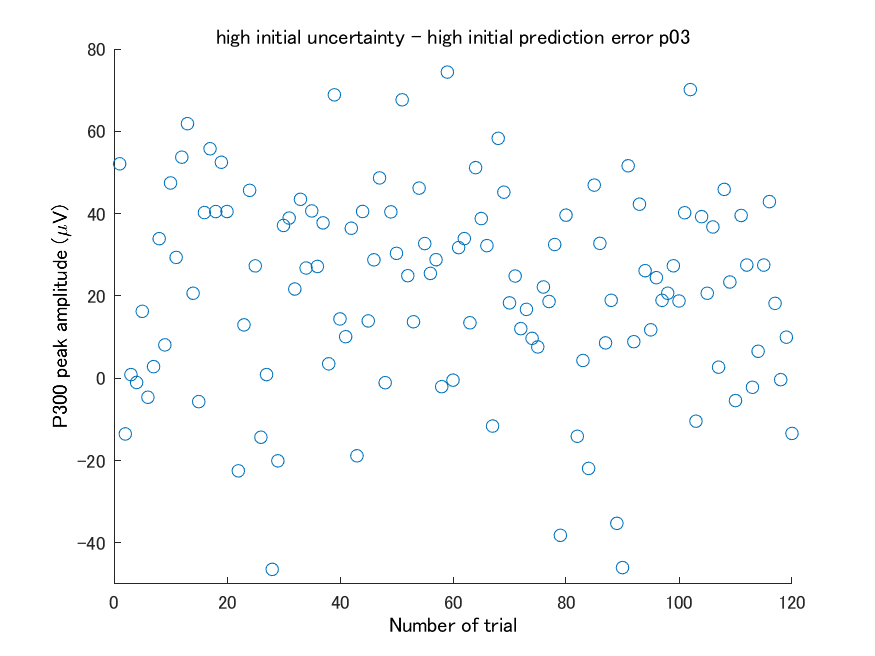

Supplement: S1 File — (ZIP) [file pone.0237278.s001.zip › supplementary material/high initial uncertainty_high initial prediction error_p03.tif]

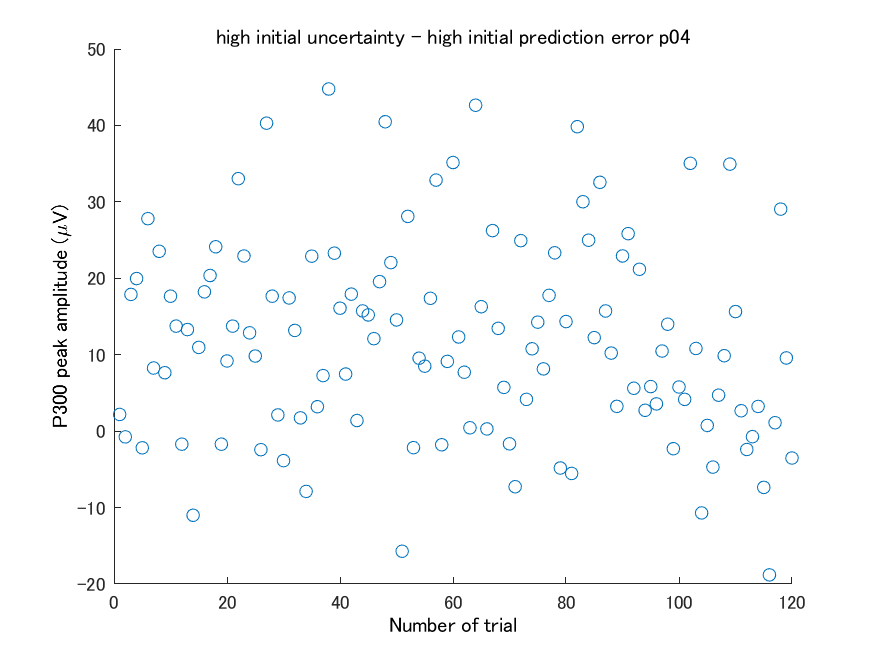

Supplement: S1 File — (ZIP) [file pone.0237278.s001.zip › supplementary material/high initial uncertainty_high initial prediction error_p04.tif]

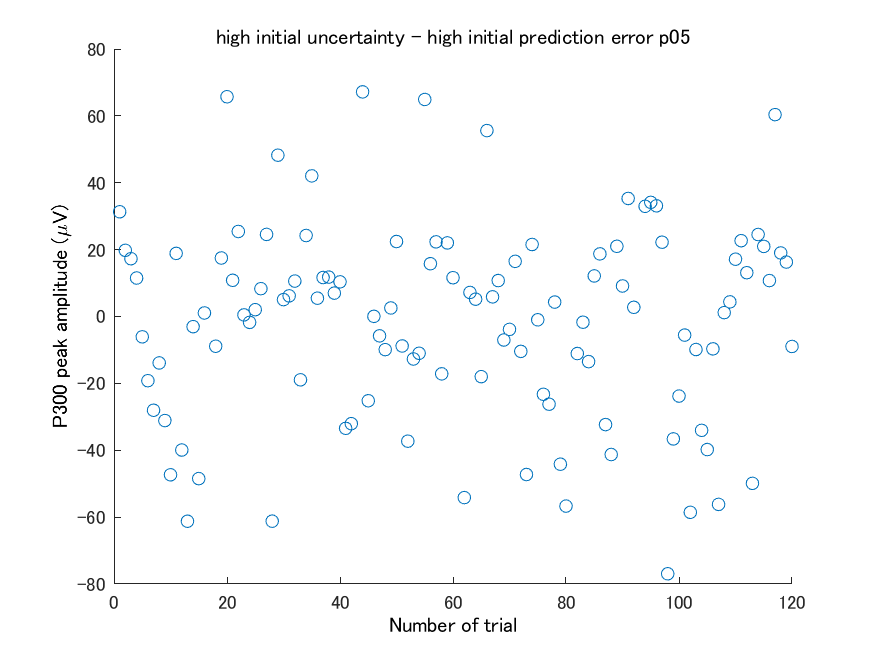

Supplement: S1 File — (ZIP) [file pone.0237278.s001.zip › supplementary material/high initial uncertainty_high initial prediction error_p05.tif]

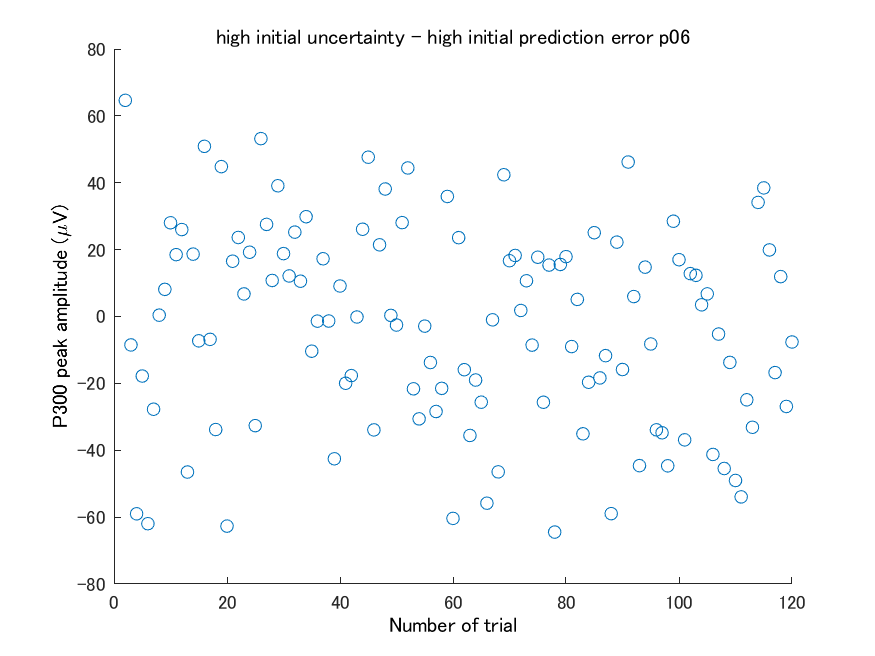

Supplement: S1 File — (ZIP) [file pone.0237278.s001.zip › supplementary material/high initial uncertainty_high initial prediction error_p06.tif]

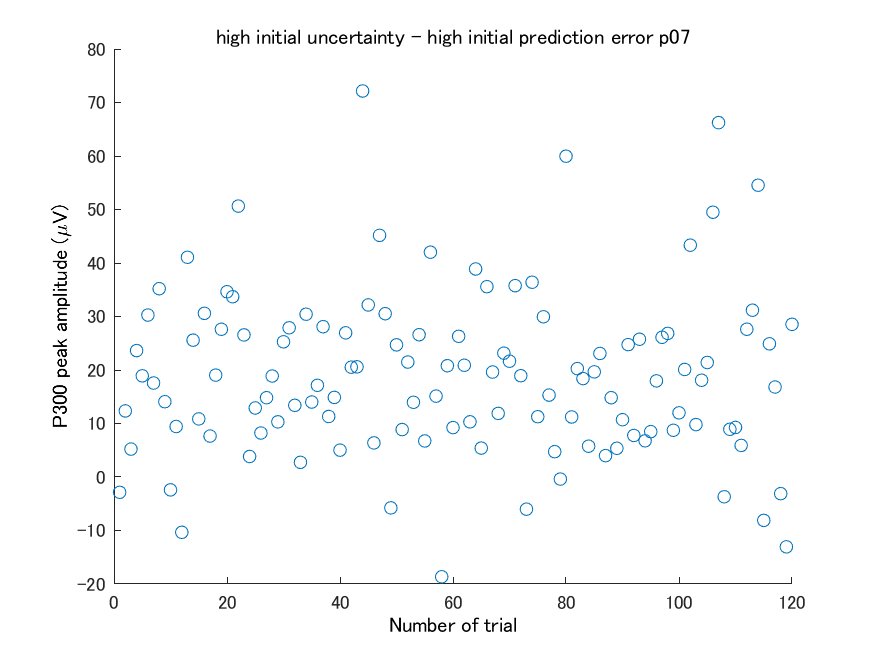

Supplement: S1 File — (ZIP) [file pone.0237278.s001.zip › supplementary material/high initial uncertainty_high initial prediction error_p07.tif]

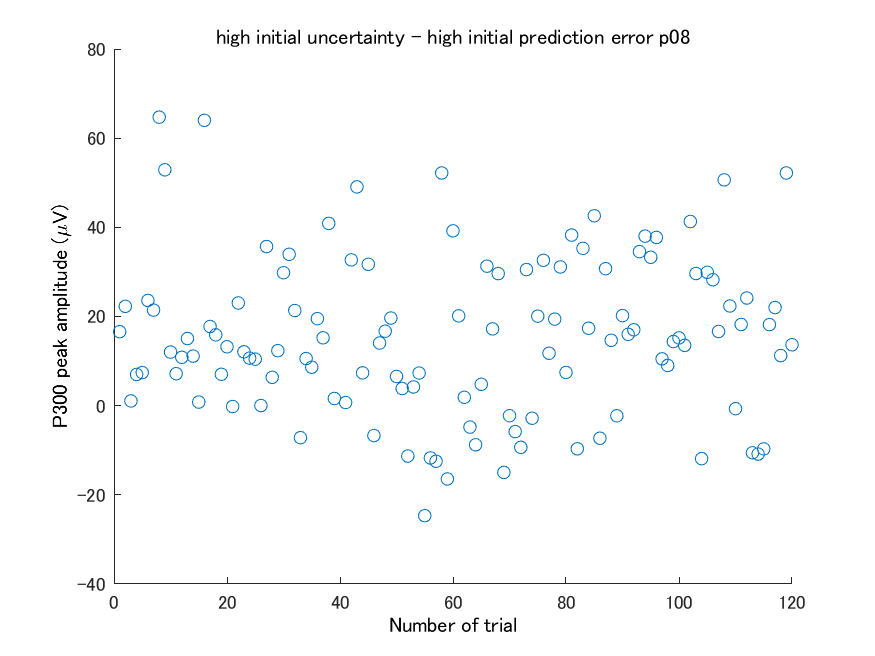

Supplement: S1 File — (ZIP) [file pone.0237278.s001.zip › supplementary material/high initial uncertainty_high initial prediction error_p08.tif]

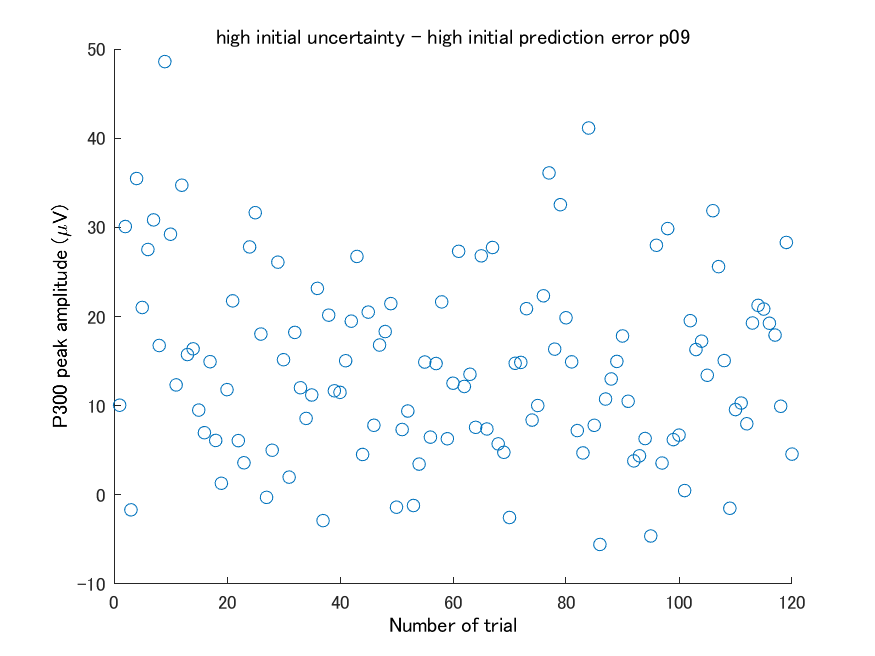

Supplement: S1 File — (ZIP) [file pone.0237278.s001.zip › supplementary material/high initial uncertainty_high initial prediction error_p09.tif]

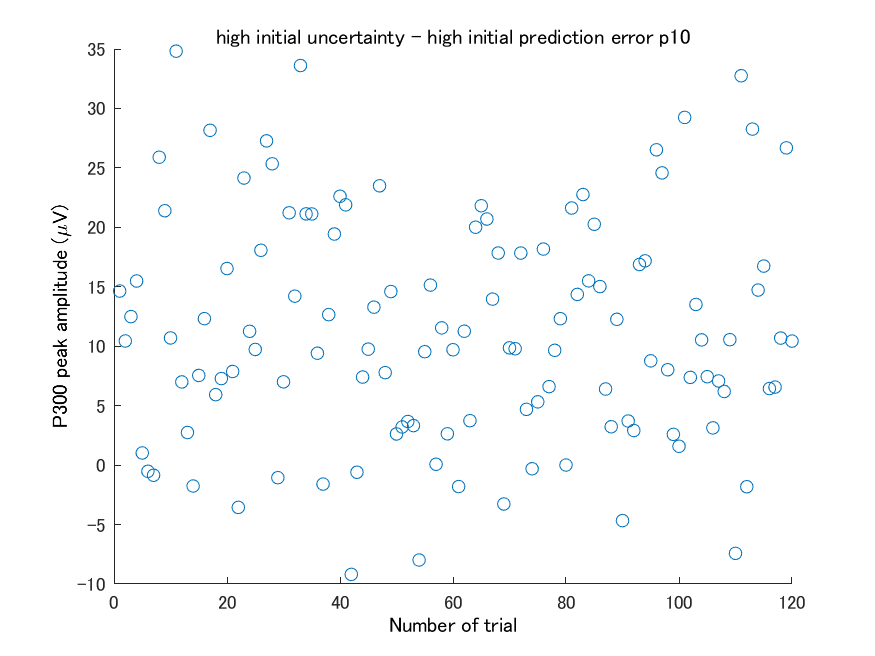

Supplement: S1 File — (ZIP) [file pone.0237278.s001.zip › supplementary material/high initial uncertainty_high initial prediction error_p10.tif]

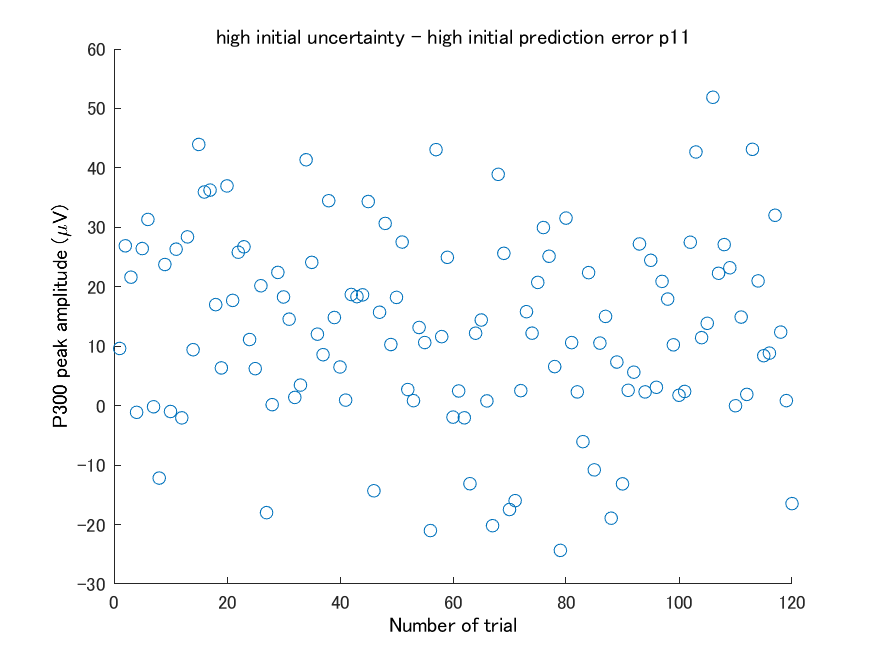

Supplement: S1 File — (ZIP) [file pone.0237278.s001.zip › supplementary material/high initial uncertainty_high initial prediction error_p11.tif]

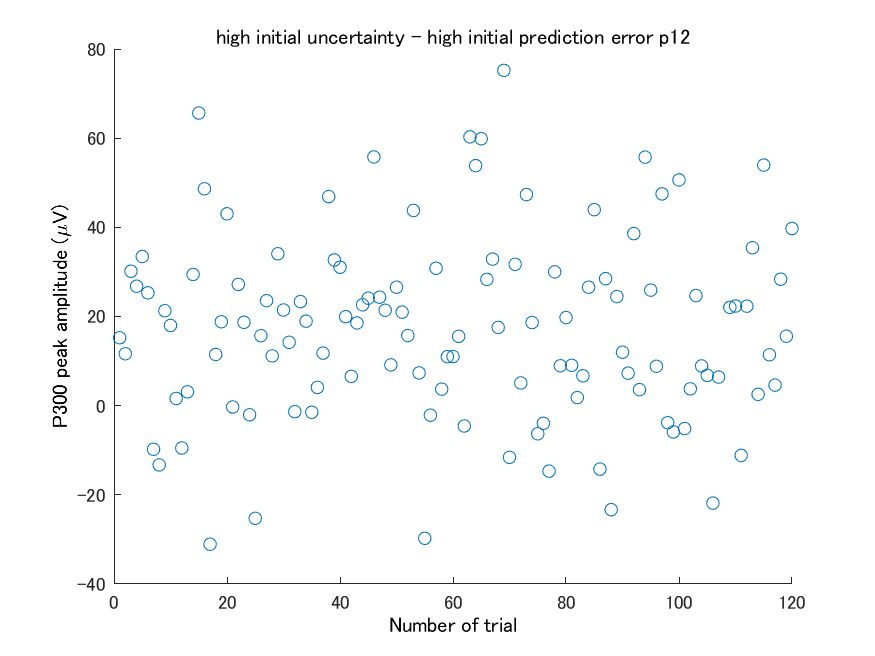

Supplement: S1 File — (ZIP) [file pone.0237278.s001.zip › supplementary material/high initial uncertainty_high initial prediction error_p12.tif]

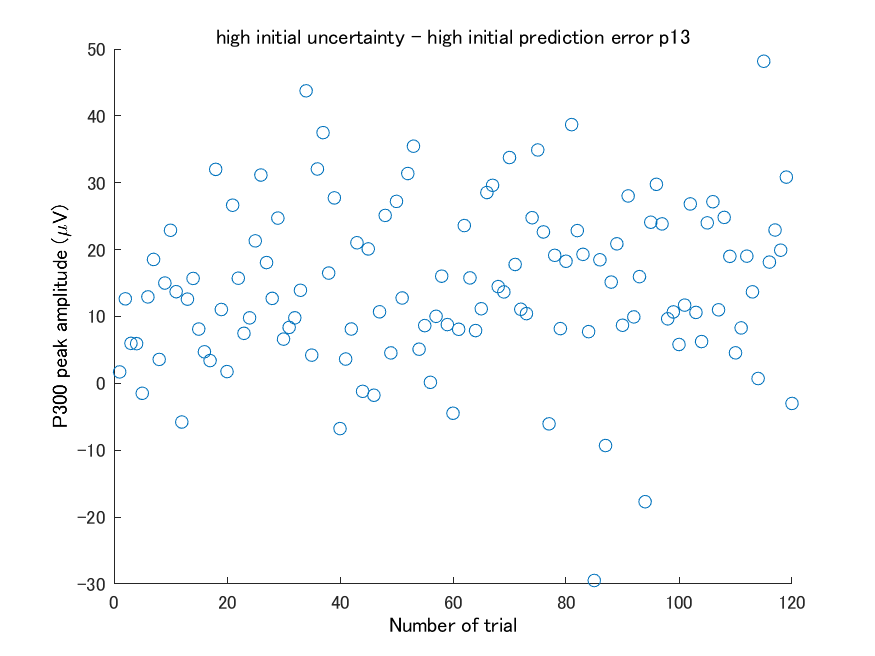

Supplement: S1 File — (ZIP) [file pone.0237278.s001.zip › supplementary material/high initial uncertainty_high initial prediction error_p13.tif]

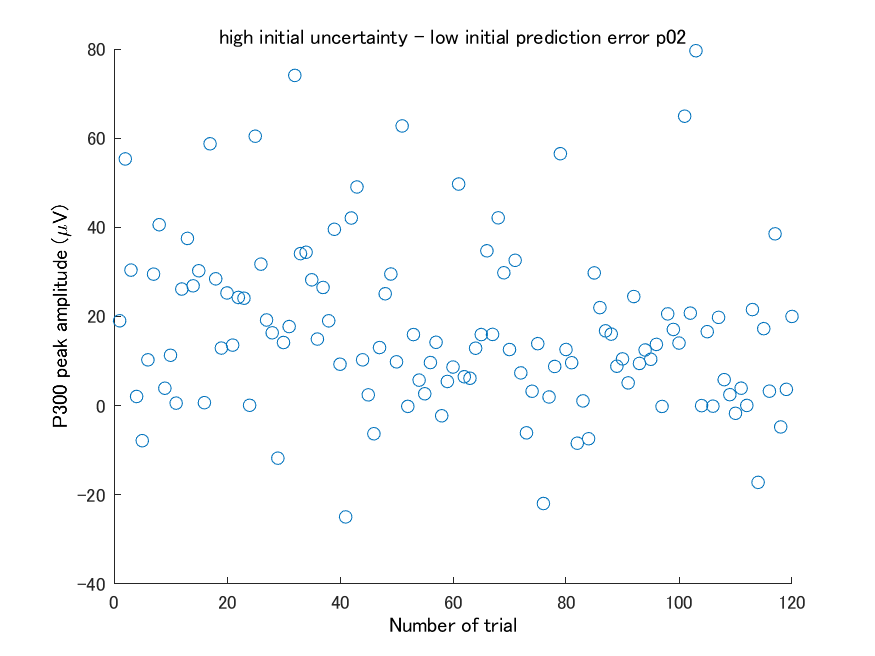

Supplement: S1 File — (ZIP) [file pone.0237278.s001.zip › supplementary material/high initial uncertainty_low initial prediction error_p02.tif]

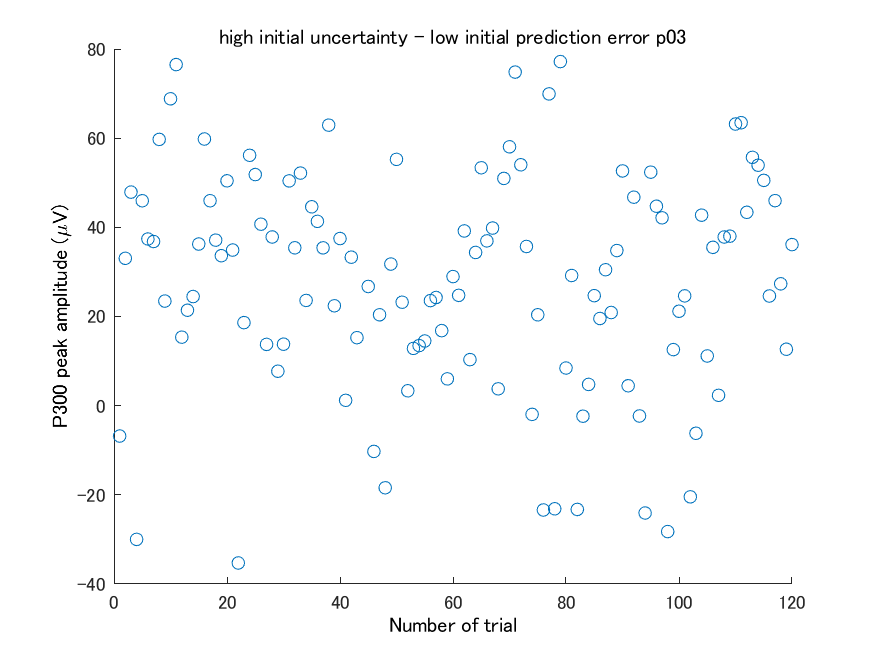

Supplement: S1 File — (ZIP) [file pone.0237278.s001.zip › supplementary material/high initial uncertainty_low initial prediction error_p03.tif]

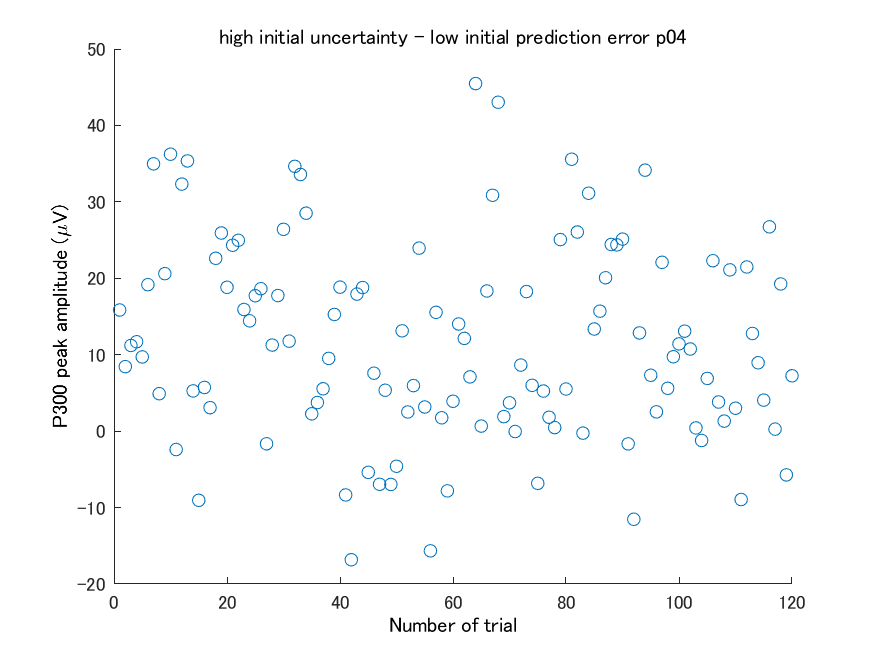

Supplement: S1 File — (ZIP) [file pone.0237278.s001.zip › supplementary material/high initial uncertainty_low initial prediction error_p04.tif]

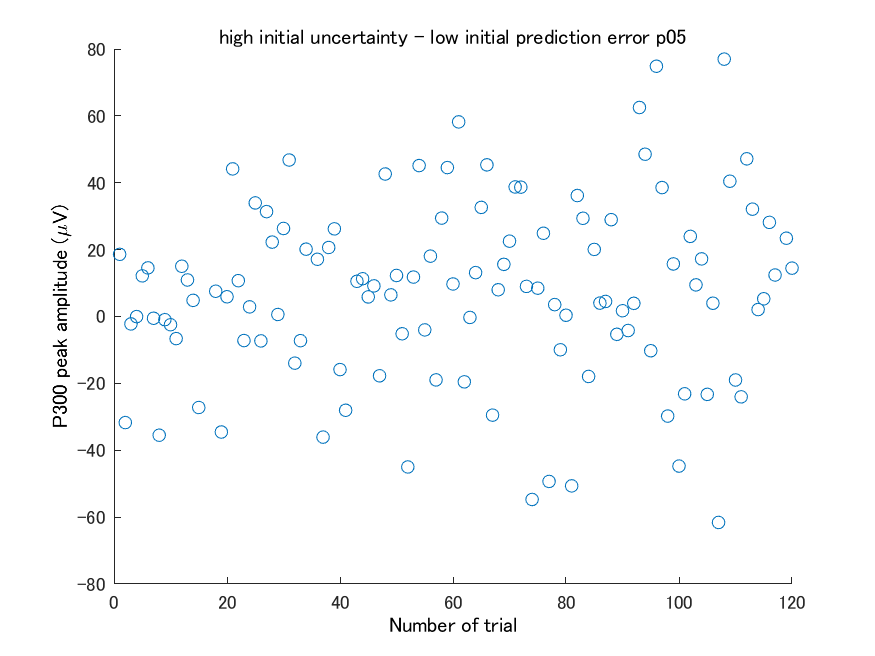

Supplement: S1 File — (ZIP) [file pone.0237278.s001.zip › supplementary material/high initial uncertainty_low initial prediction error_p05.tif]

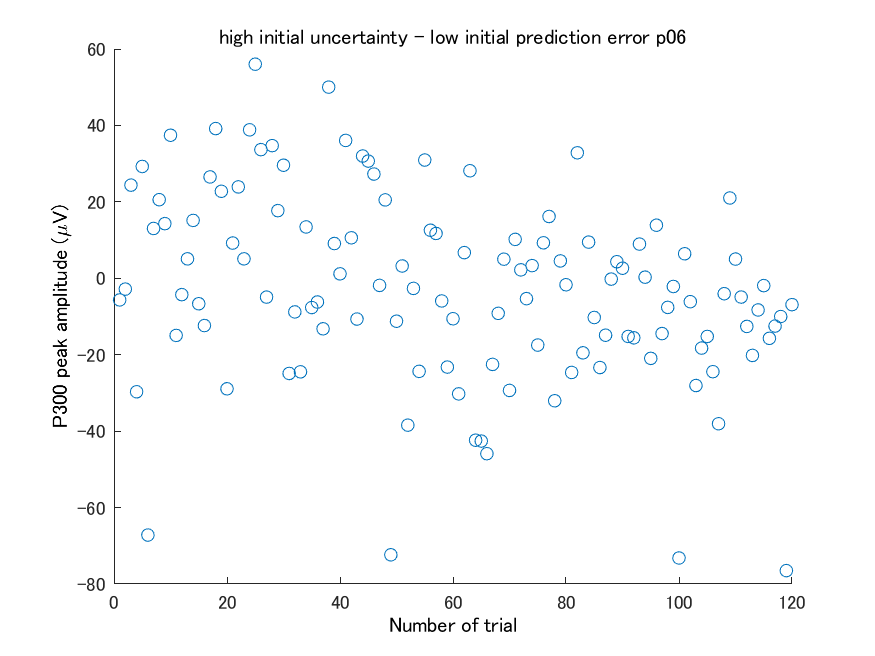

Supplement: S1 File — (ZIP) [file pone.0237278.s001.zip › supplementary material/high initial uncertainty_low initial prediction error_p06.tif]

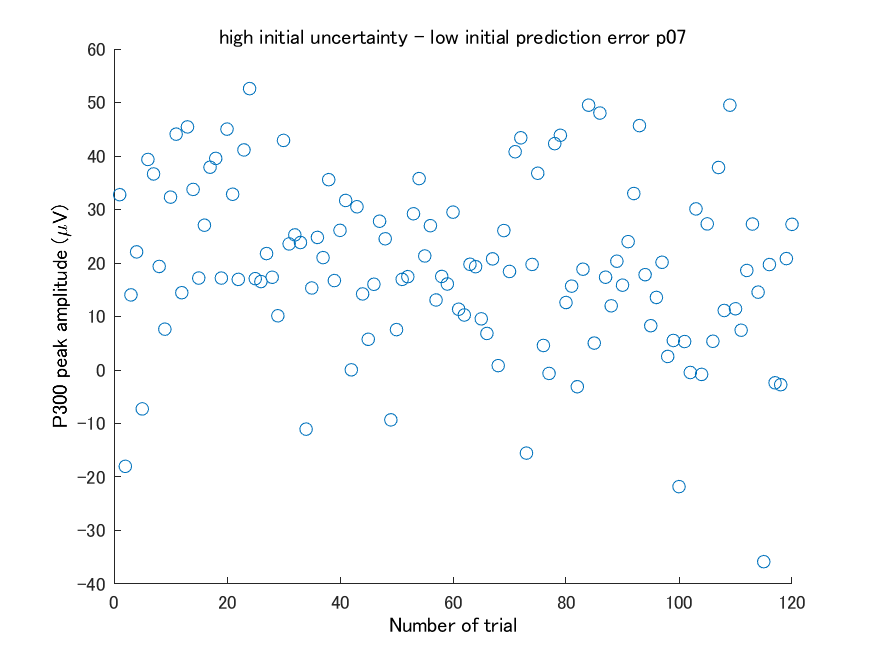

Supplement: S1 File — (ZIP) [file pone.0237278.s001.zip › supplementary material/high initial uncertainty_low initial prediction error_p07.tif]

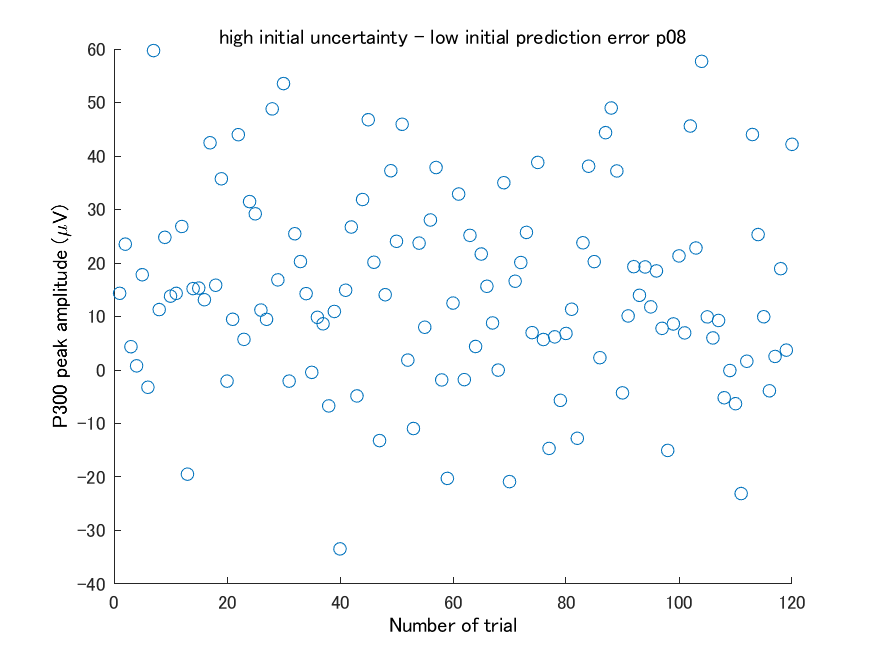

Supplement: S1 File — (ZIP) [file pone.0237278.s001.zip › supplementary material/high initial uncertainty_low initial prediction error_p08.tif]

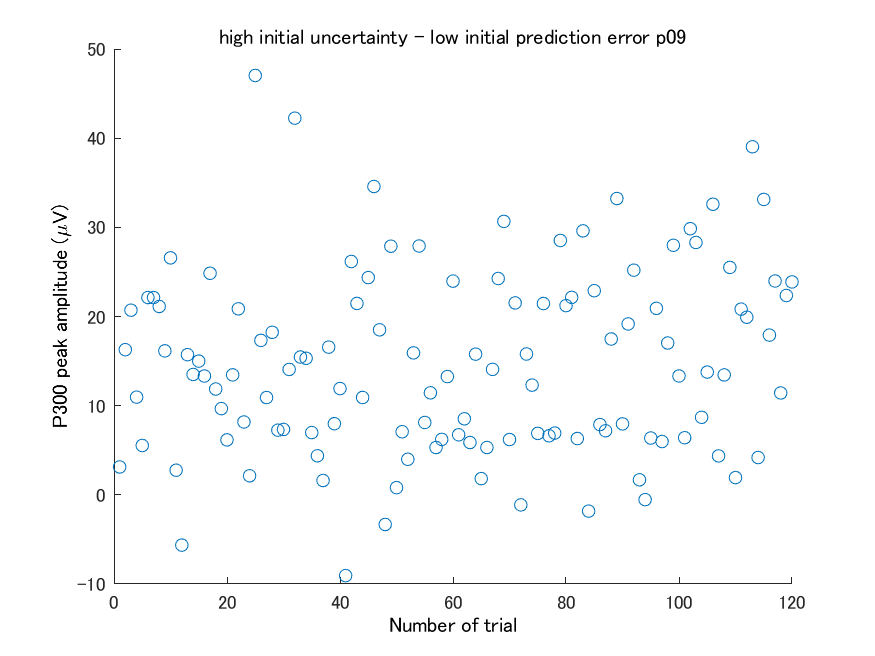

Supplement: S1 File — (ZIP) [file pone.0237278.s001.zip › supplementary material/high initial uncertainty_low initial prediction error_p09.tif]

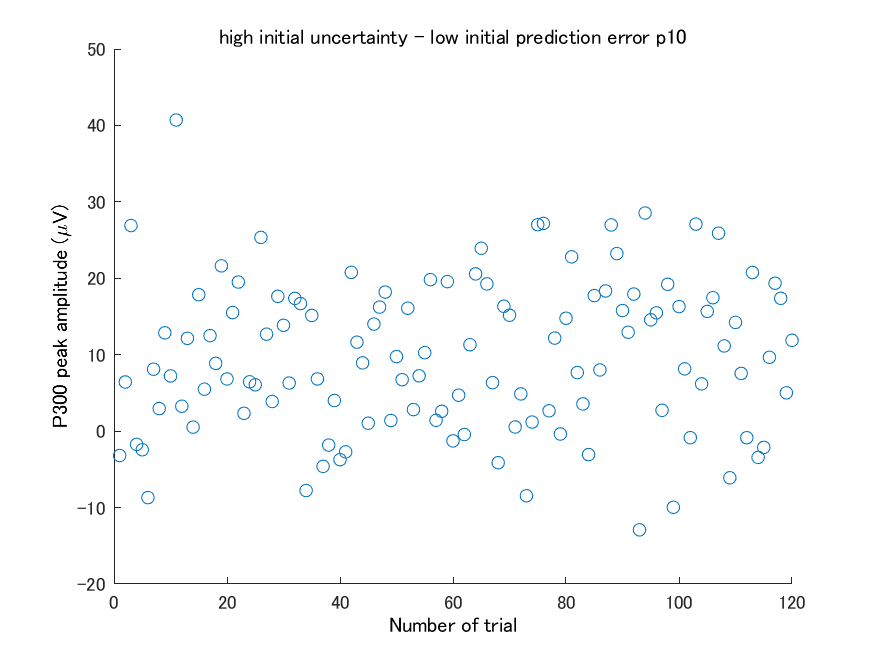

Supplement: S1 File — (ZIP) [file pone.0237278.s001.zip › supplementary material/high initial uncertainty_low initial prediction error_p10.tif]

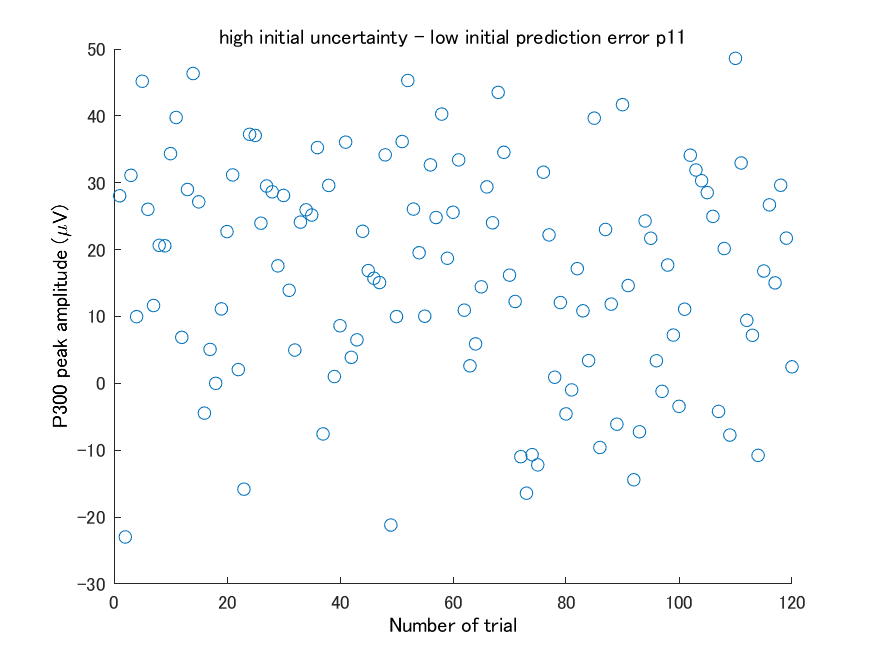

Supplement: S1 File — (ZIP) [file pone.0237278.s001.zip › supplementary material/high initial uncertainty_low initial prediction error_p11.tif]

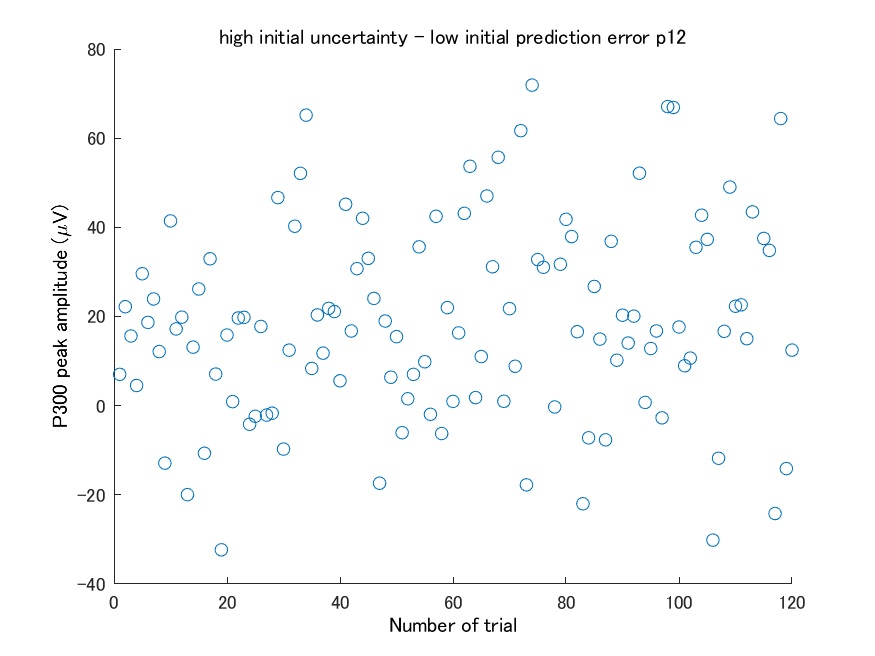

Supplement: S1 File — (ZIP) [file pone.0237278.s001.zip › supplementary material/high initial uncertainty_low initial prediction error_p12.tif]

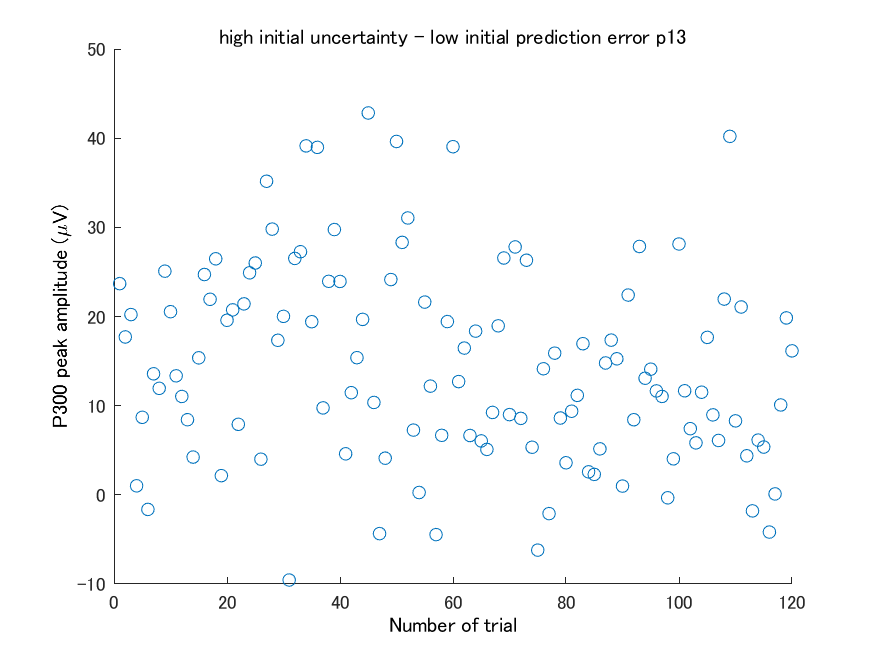

Supplement: S1 File — (ZIP) [file pone.0237278.s001.zip › supplementary material/high initial uncertainty_low initial prediction error_p13.tif]

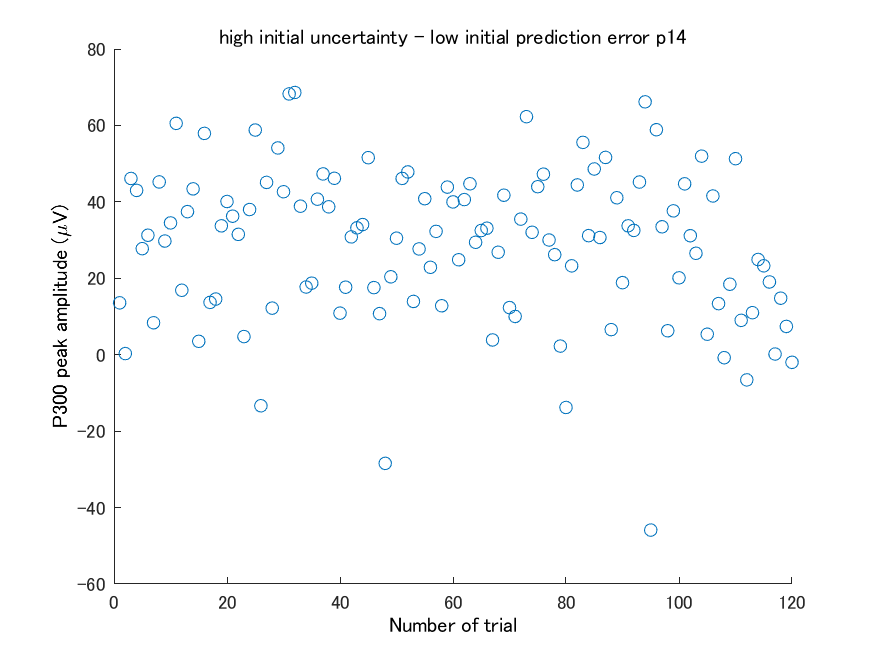

Supplement: S1 File — (ZIP) [file pone.0237278.s001.zip › supplementary material/high initial uncertainty_low initial prediction error_p14.tif]

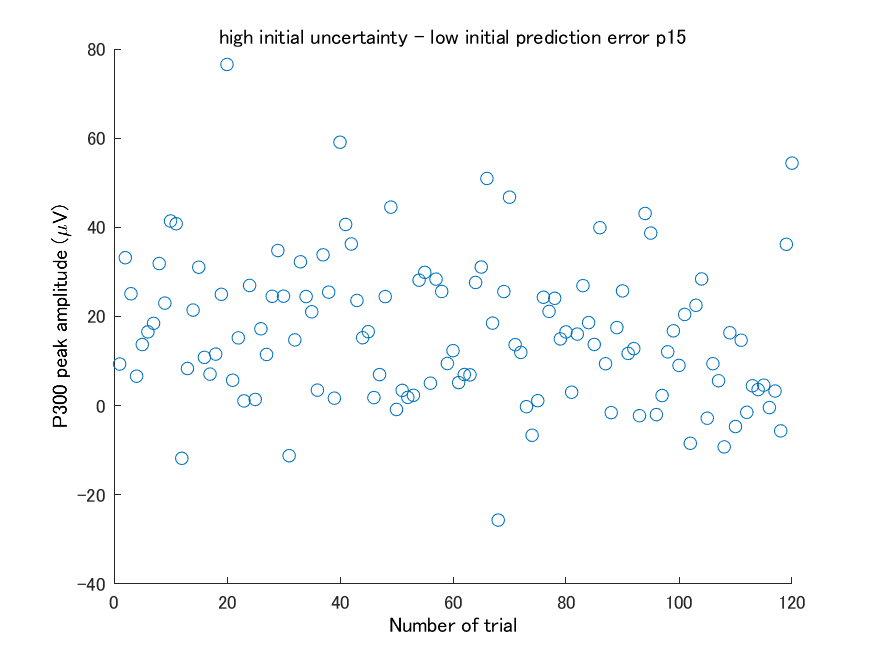

Supplement: S1 File — (ZIP) [file pone.0237278.s001.zip › supplementary material/high initial uncertainty_low initial prediction error_p15.tif]

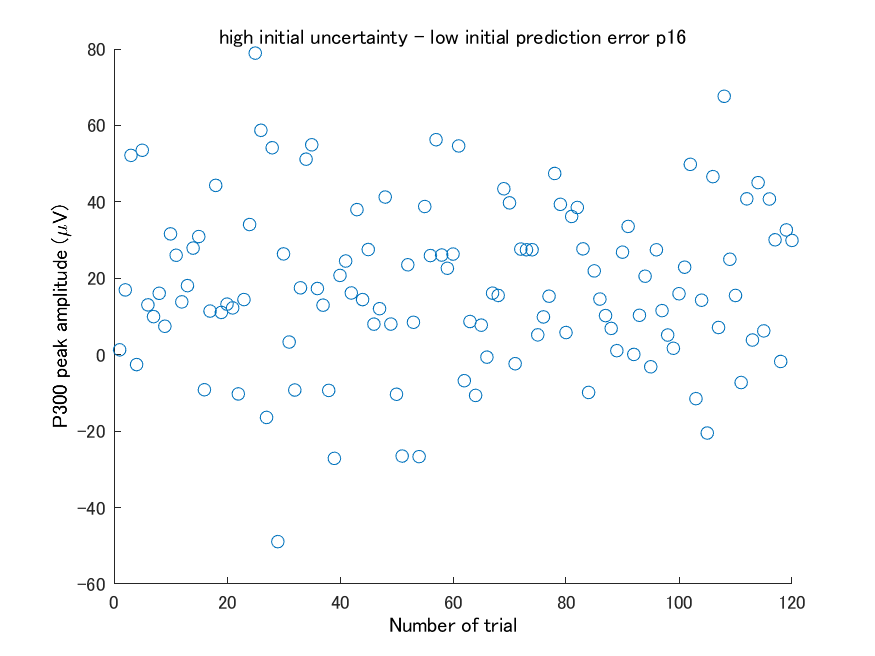

Supplement: S1 File — (ZIP) [file pone.0237278.s001.zip › supplementary material/high initial uncertainty_low initial prediction error_p16.tif]

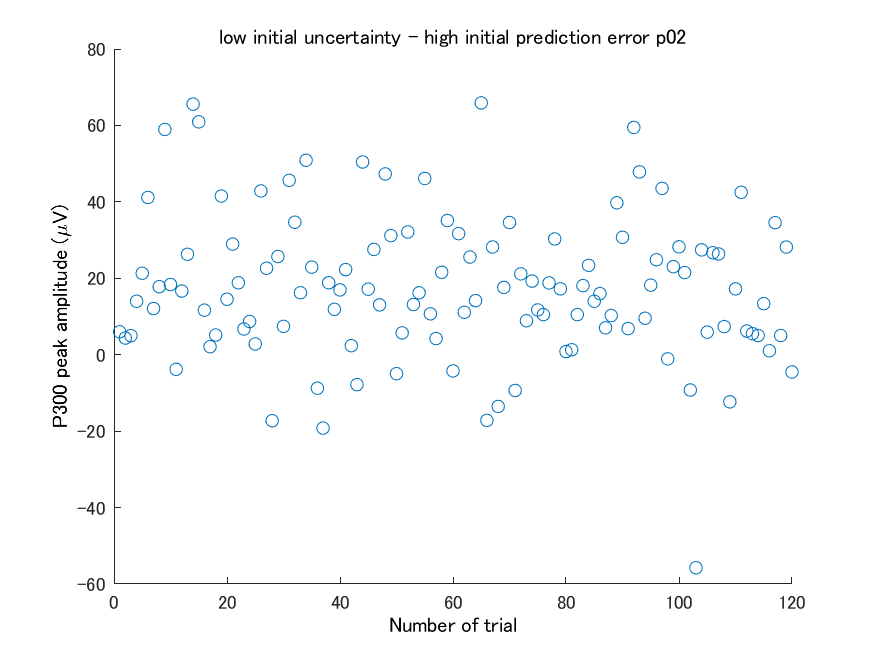

Supplement: S1 File — (ZIP) [file pone.0237278.s001.zip › supplementary material/low initial uncertainty_high initial prediction error_p02.tif]

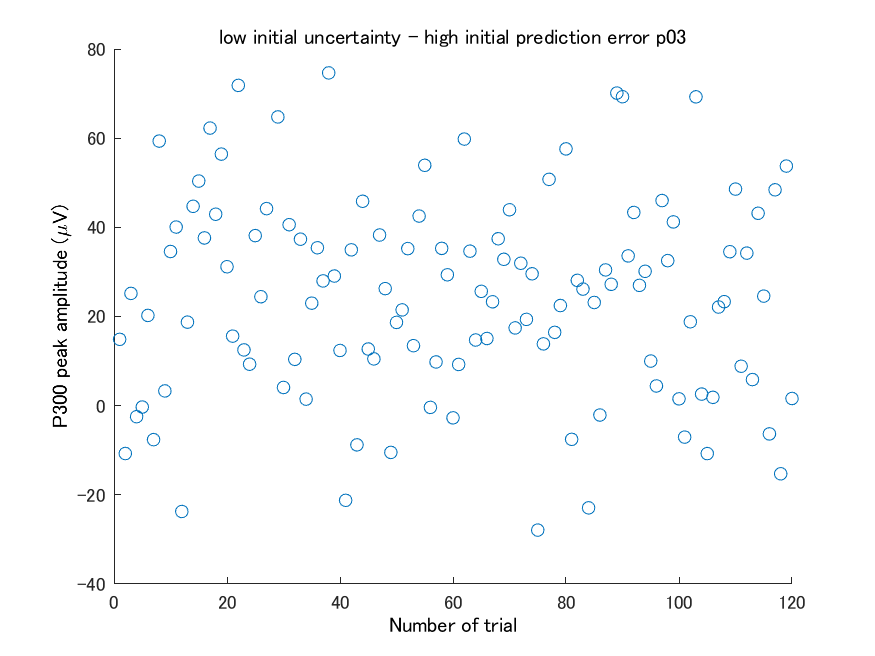

Supplement: S1 File — (ZIP) [file pone.0237278.s001.zip › supplementary material/low initial uncertainty_high initial prediction error_p03.tif]

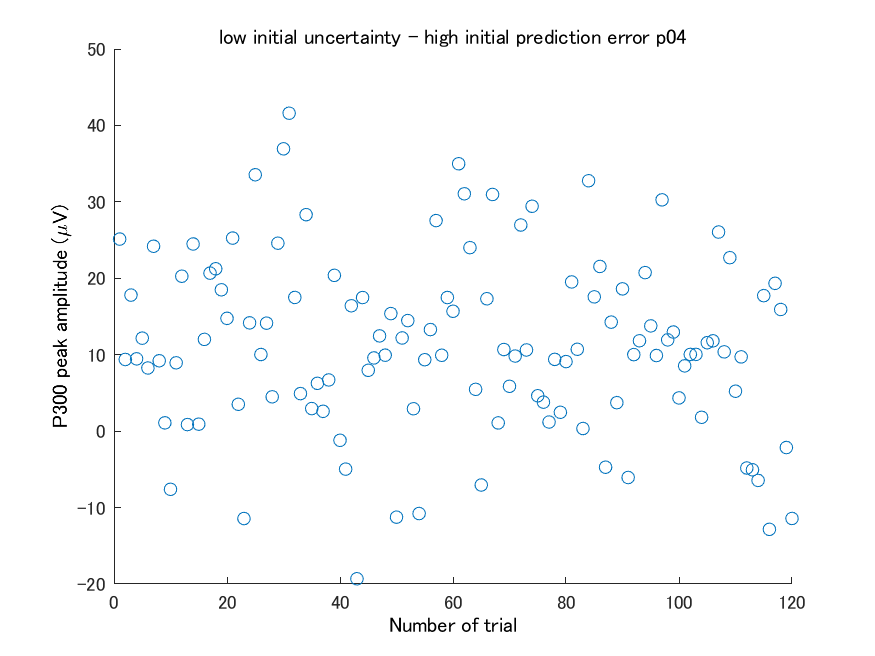

Supplement: S1 File — (ZIP) [file pone.0237278.s001.zip › supplementary material/low initial uncertainty_high initial prediction error_p04.tif]

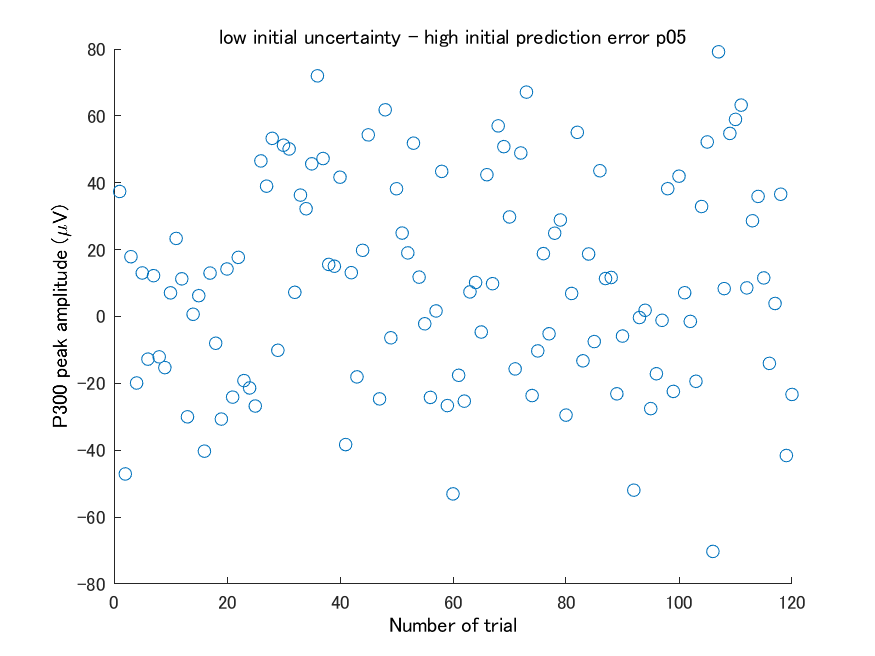

Supplement: S1 File — (ZIP) [file pone.0237278.s001.zip › supplementary material/low initial uncertainty_high initial prediction error_p05.tif]

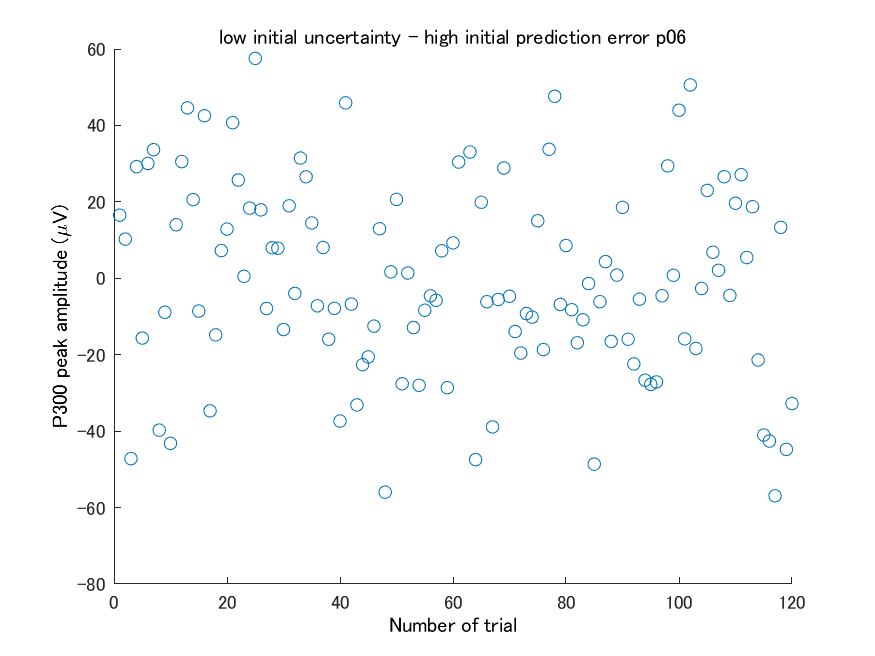

Supplement: S1 File — (ZIP) [file pone.0237278.s001.zip › supplementary material/low initial uncertainty_high initial prediction error_p06.tif]

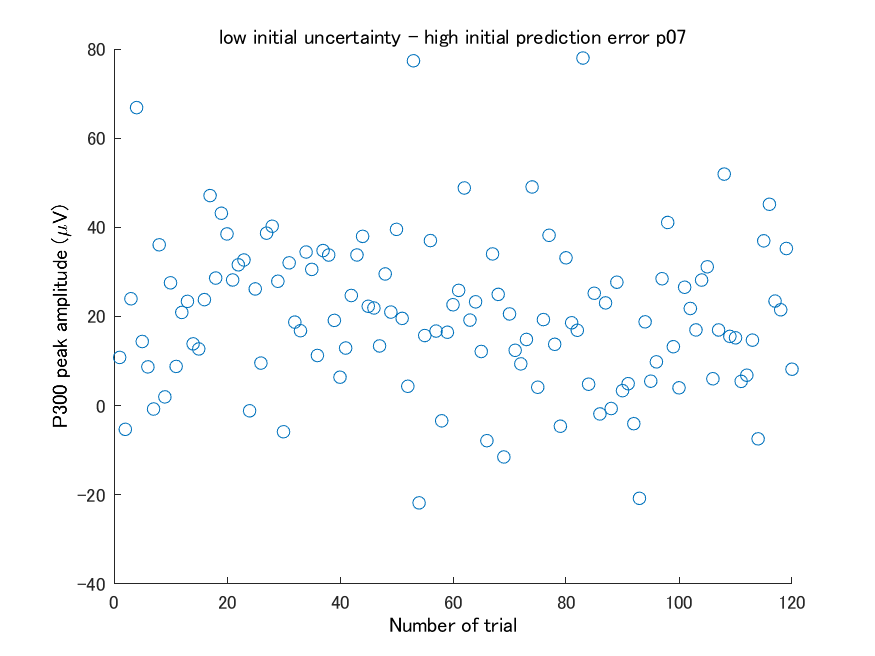

Supplement: S1 File — (ZIP) [file pone.0237278.s001.zip › supplementary material/low initial uncertainty_high initial prediction error_p07.tif]

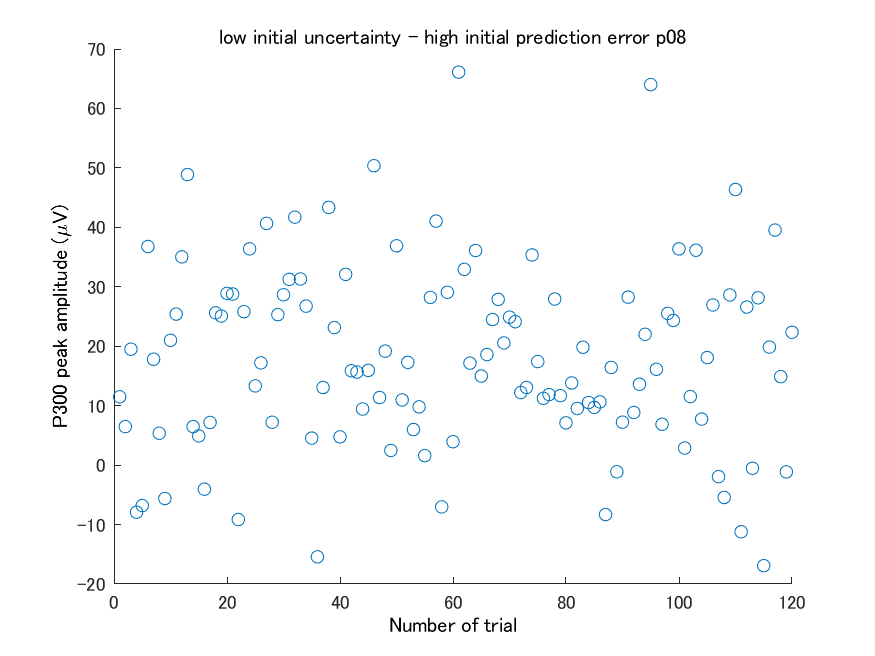

Supplement: S1 File — (ZIP) [file pone.0237278.s001.zip › supplementary material/low initial uncertainty_high initial prediction error_p08.tif]

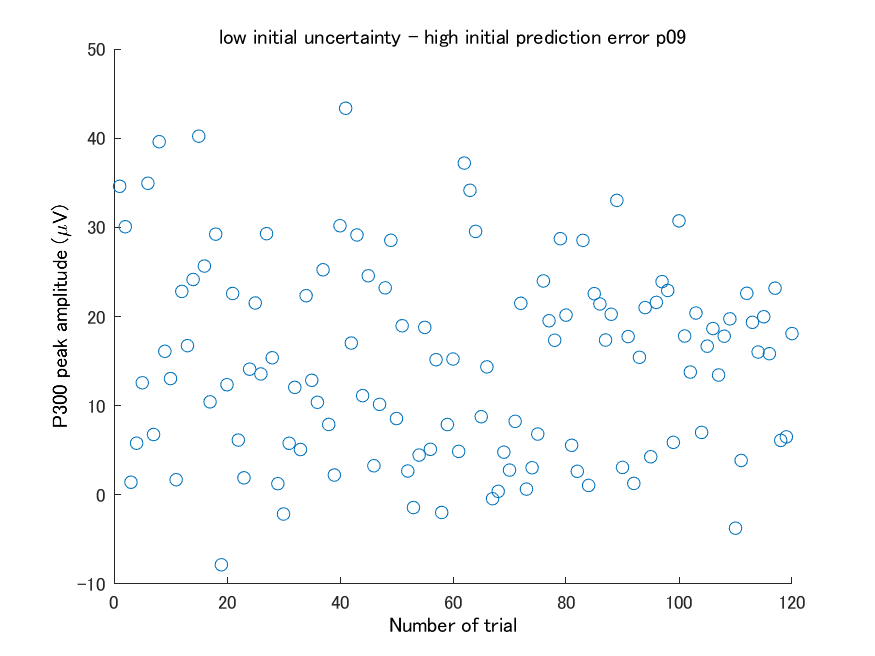

Supplement: S1 File — (ZIP) [file pone.0237278.s001.zip › supplementary material/low initial uncertainty_high initial prediction error_p09.tif]

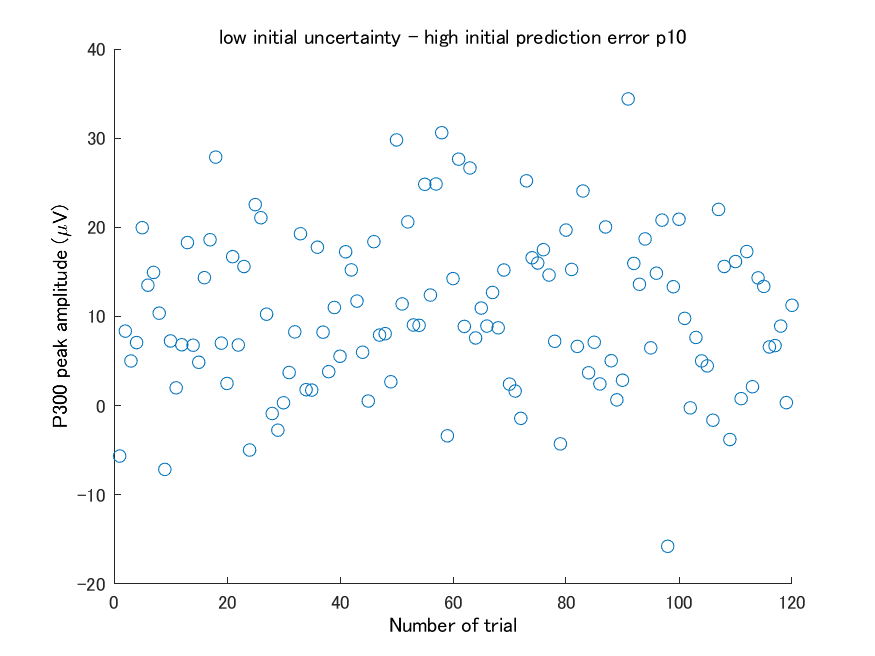

Supplement: S1 File — (ZIP) [file pone.0237278.s001.zip › supplementary material/low initial uncertainty_high initial prediction error_p10.tif]

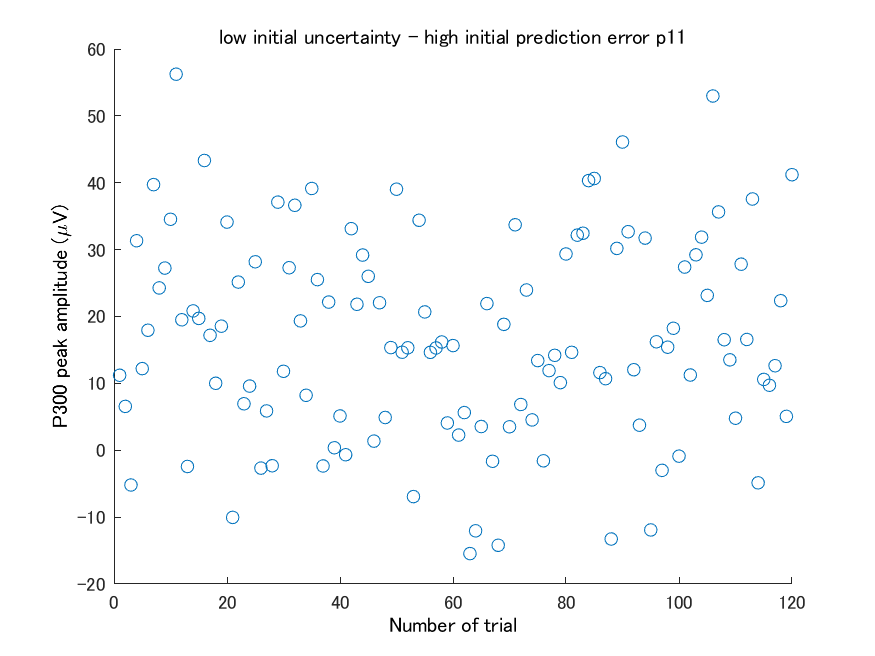

Supplement: S1 File — (ZIP) [file pone.0237278.s001.zip › supplementary material/low initial uncertainty_high initial prediction error_p11.tif]

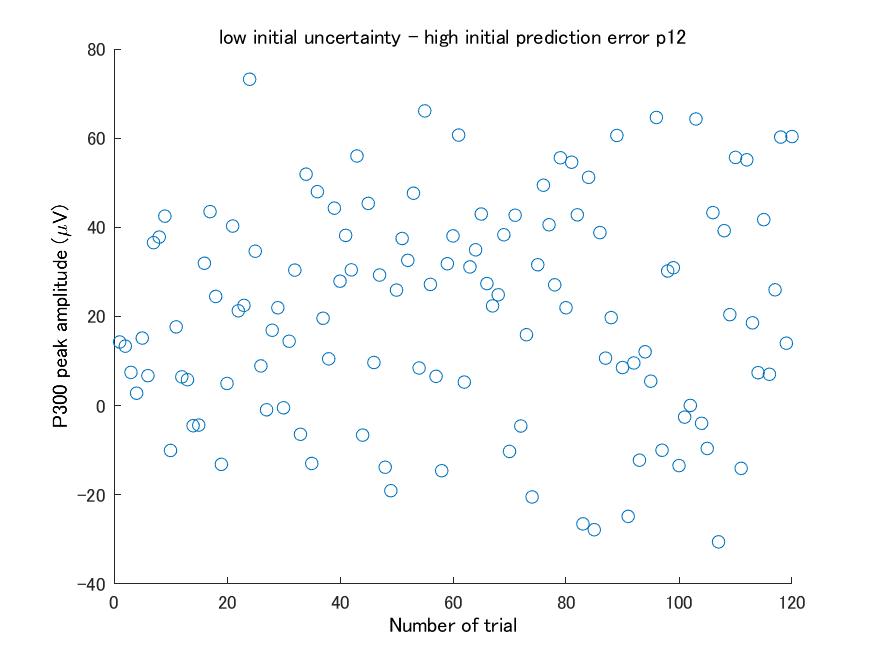

Supplement: S1 File — (ZIP) [file pone.0237278.s001.zip › supplementary material/low initial uncertainty_high initial prediction error_p12.tif]

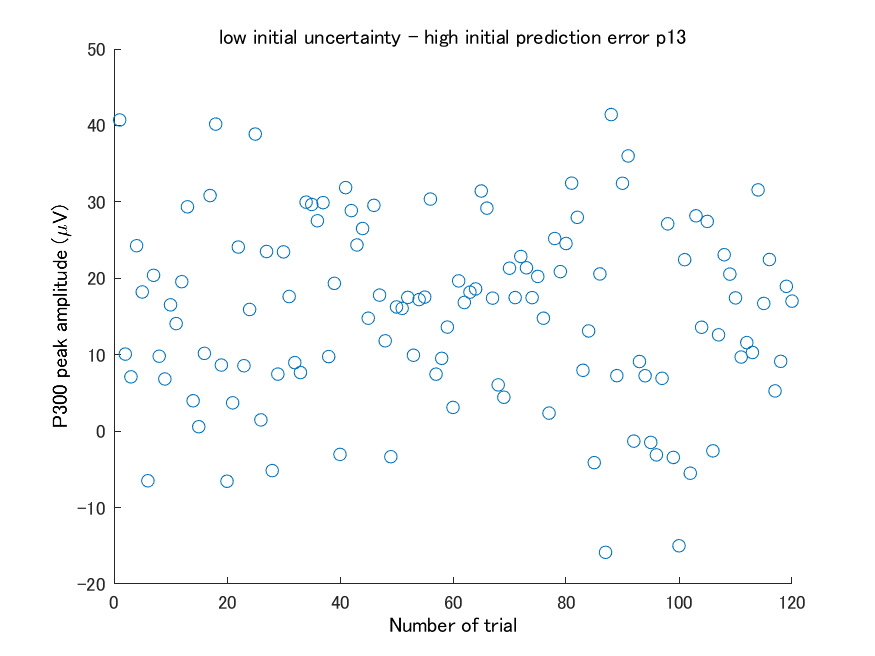

Supplement: S1 File — (ZIP) [file pone.0237278.s001.zip › supplementary material/low initial uncertainty_high initial prediction error_p13.tif]

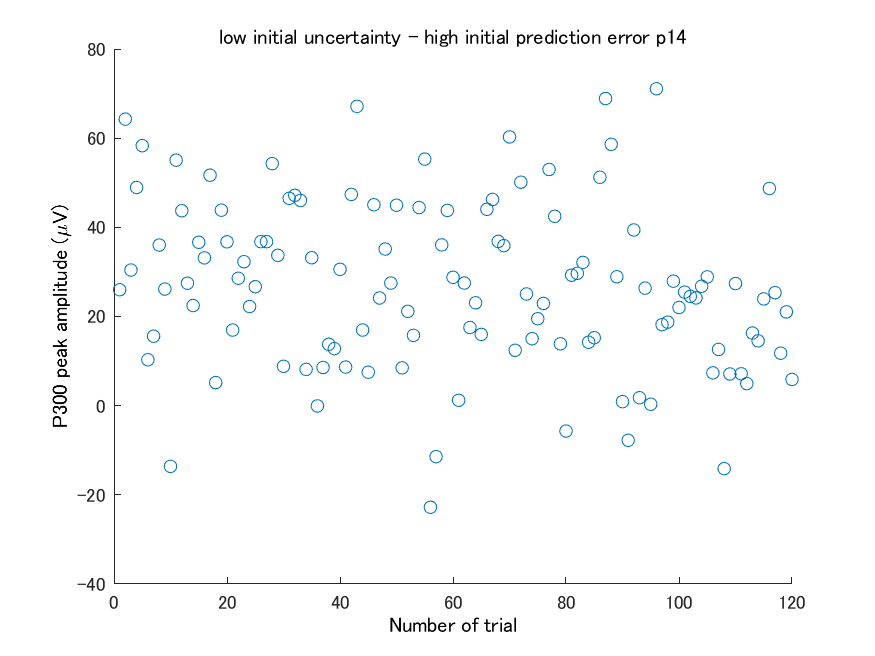

Supplement: S1 File — (ZIP) [file pone.0237278.s001.zip › supplementary material/low initial uncertainty_high initial prediction error_p14.tif]

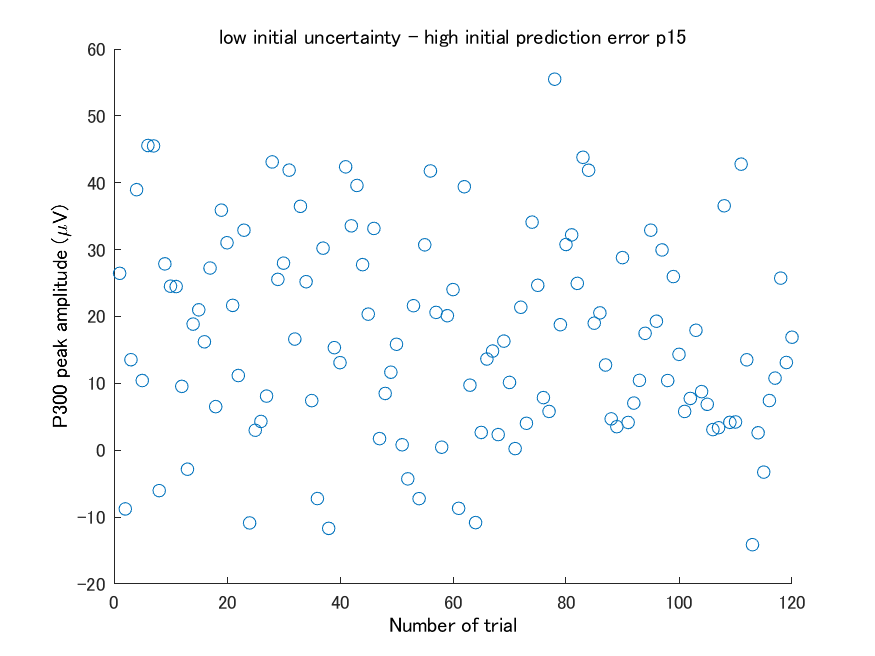

Supplement: S1 File — (ZIP) [file pone.0237278.s001.zip › supplementary material/low initial uncertainty_high initial prediction error_p15.tif]

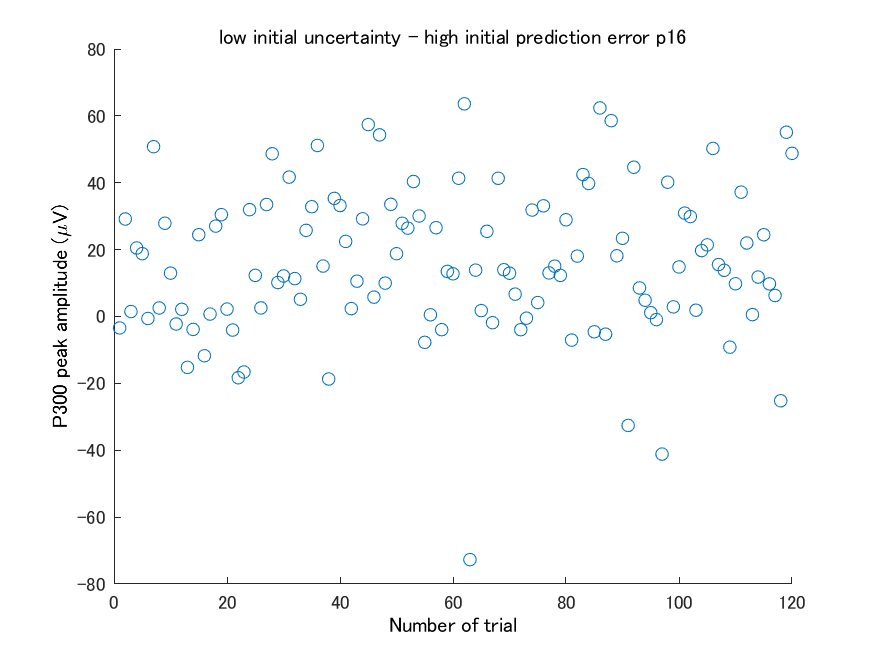

Supplement: S1 File — (ZIP) [file pone.0237278.s001.zip › supplementary material/low initial uncertainty_high initial prediction error_p16.tif]

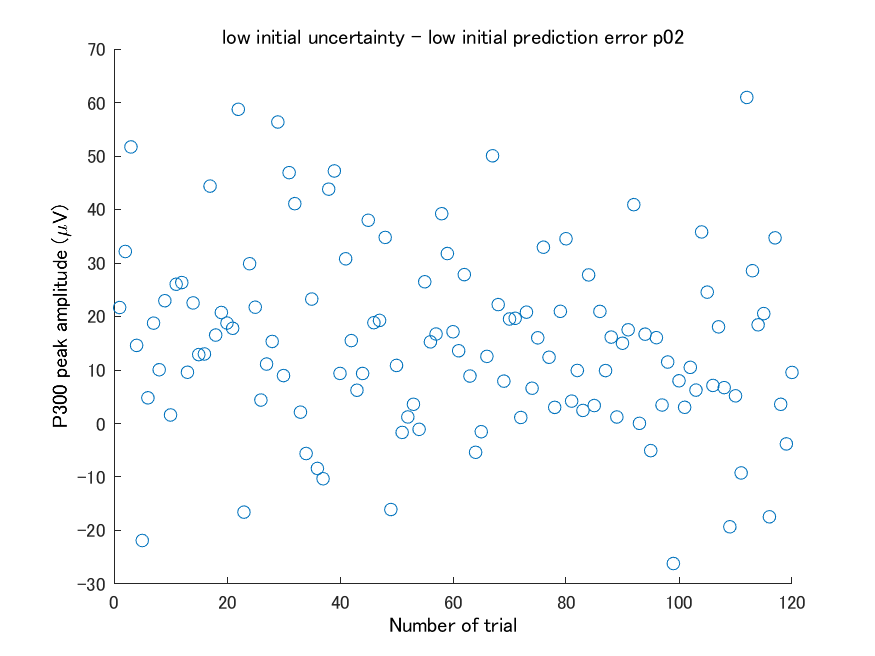

Supplement: S1 File — (ZIP) [file pone.0237278.s001.zip › supplementary material/low initial uncertainty_low initial prediction error_p02.tif]

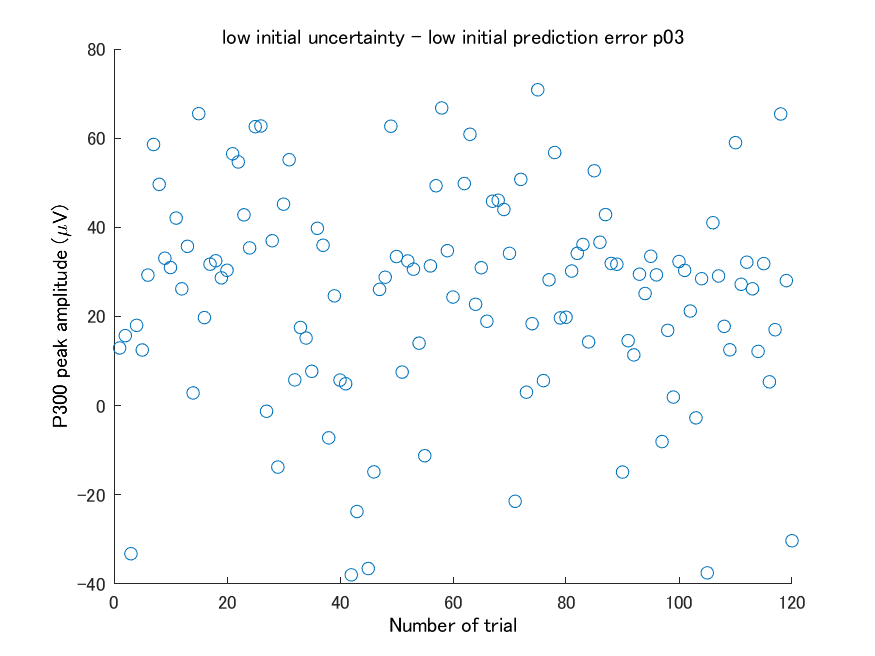

Supplement: S1 File — (ZIP) [file pone.0237278.s001.zip › supplementary material/low initial uncertainty_low initial prediction error_p03.tif]

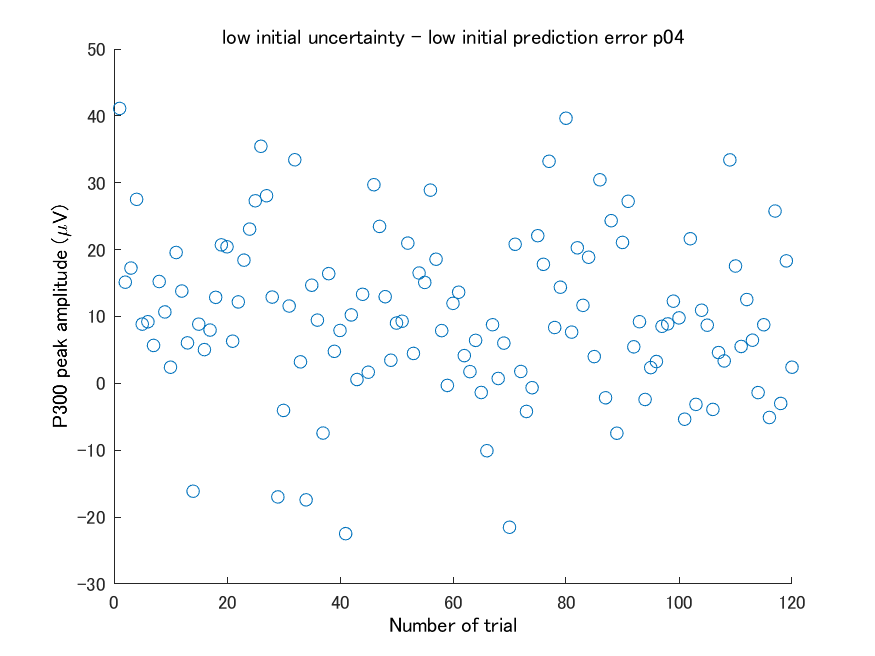

Supplement: S1 File — (ZIP) [file pone.0237278.s001.zip › supplementary material/low initial uncertainty_low initial prediction error_p04.tif]

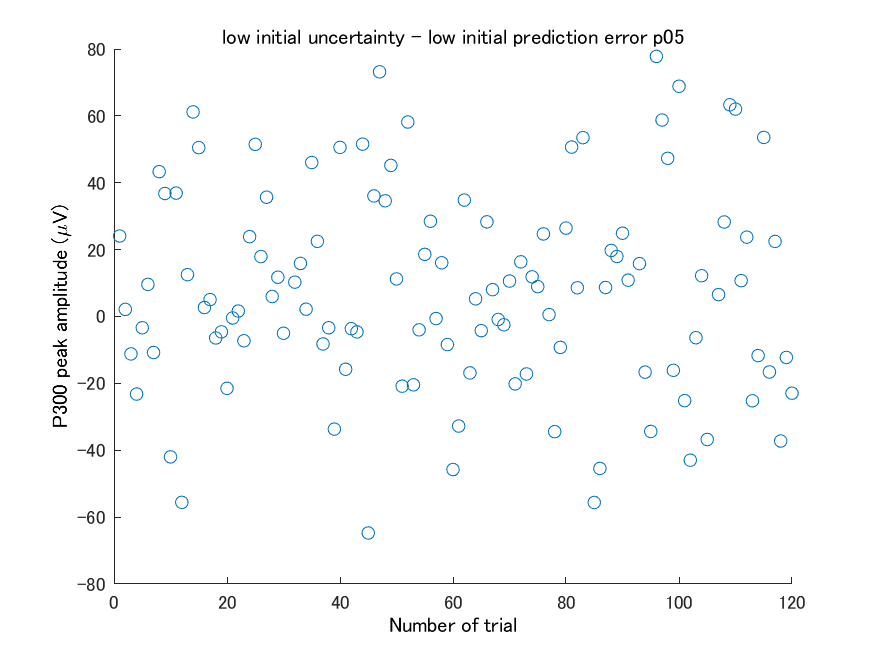

Supplement: S1 File — (ZIP) [file pone.0237278.s001.zip › supplementary material/low initial uncertainty_low initial prediction error_p05.tif]

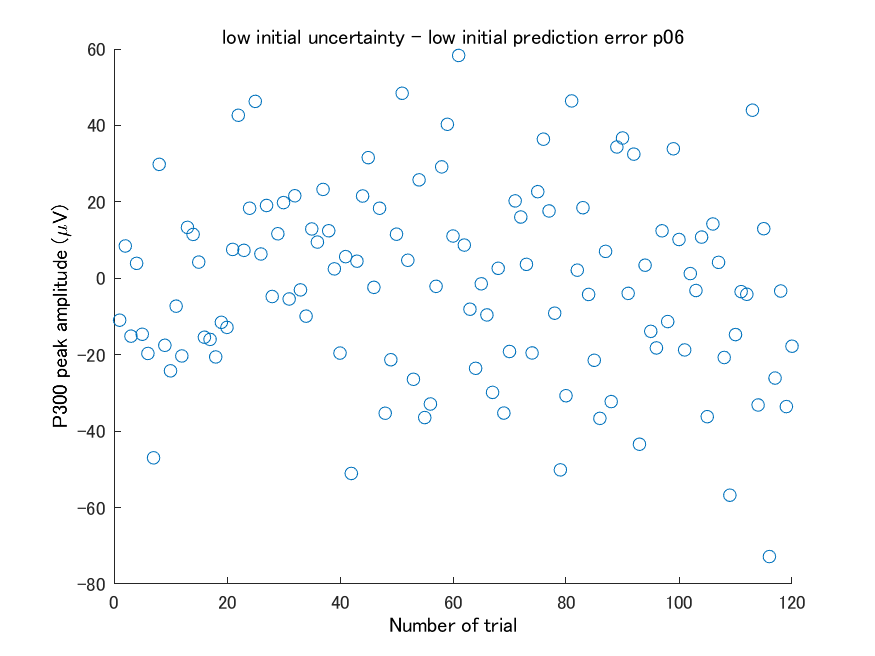

Supplement: S1 File — (ZIP) [file pone.0237278.s001.zip › supplementary material/low initial uncertainty_low initial prediction error_p06.tif]

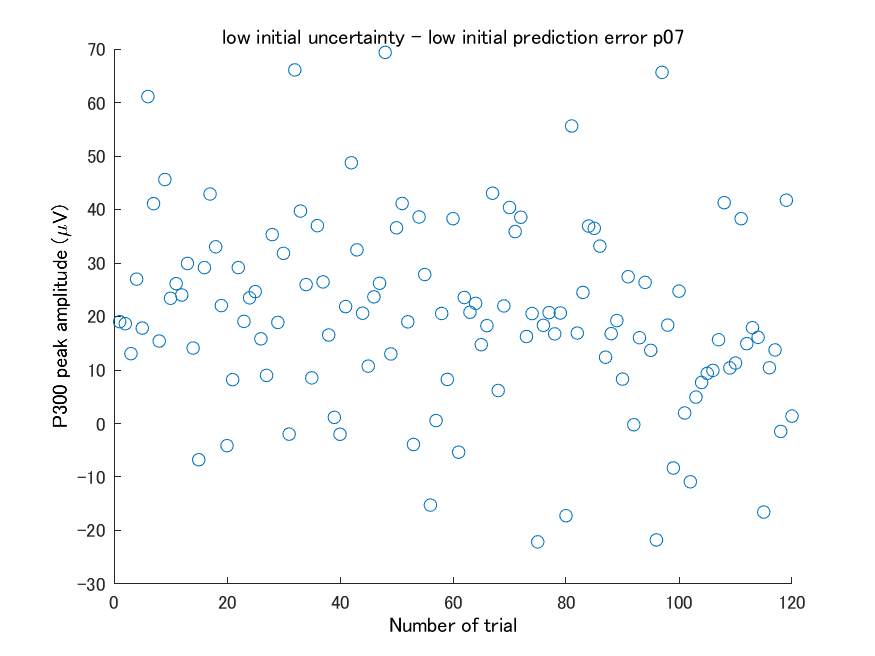

Supplement: S1 File — (ZIP) [file pone.0237278.s001.zip › supplementary material/low initial uncertainty_low initial prediction error_p07.tif]

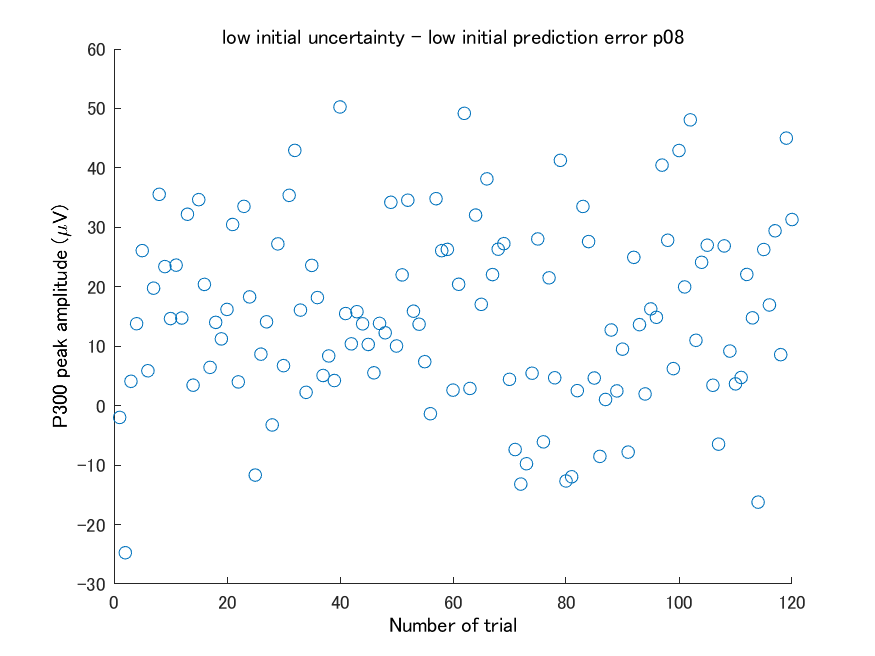

Supplement: S1 File — (ZIP) [file pone.0237278.s001.zip › supplementary material/low initial uncertainty_low initial prediction error_p08.tif]

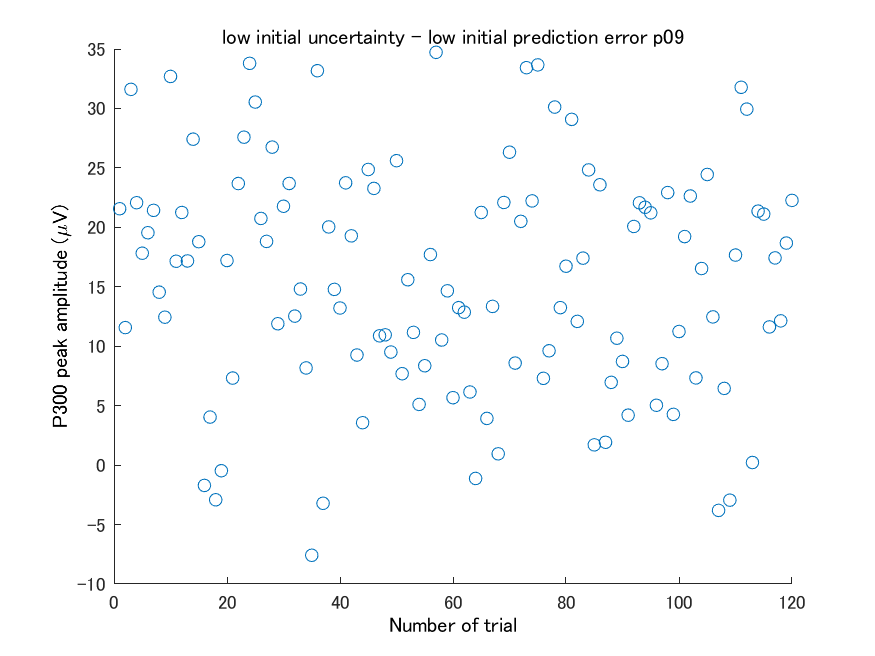

Supplement: S1 File — (ZIP) [file pone.0237278.s001.zip › supplementary material/low initial uncertainty_low initial prediction error_p09.tif]

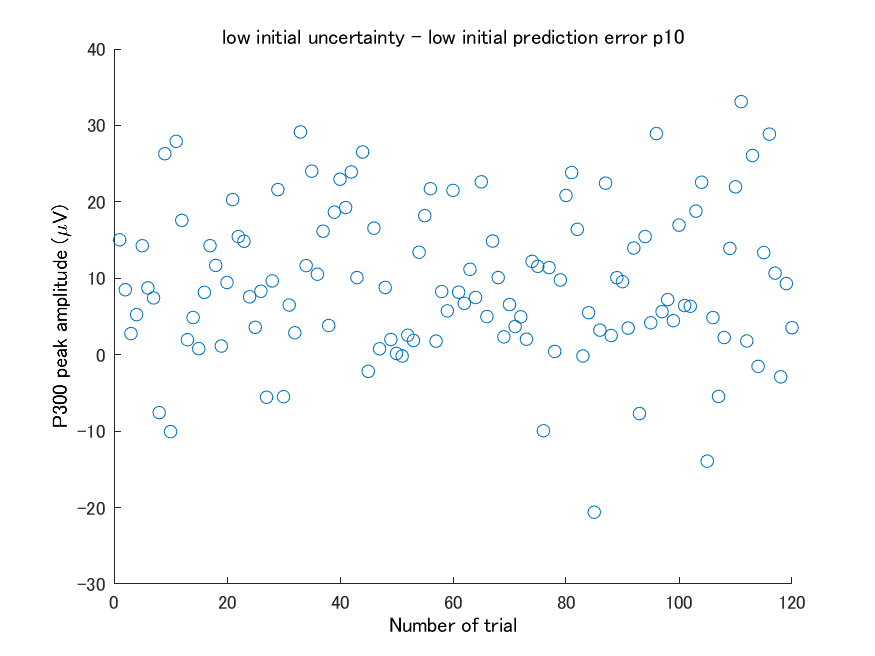

Supplement: S1 File — (ZIP) [file pone.0237278.s001.zip › supplementary material/low initial uncertainty_low initial prediction error_p10.tif]

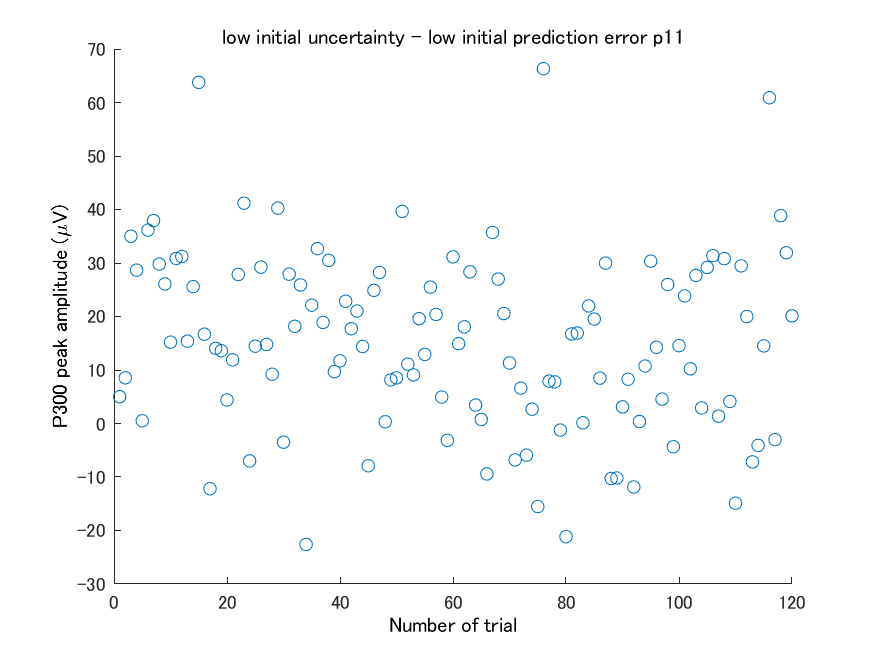

Supplement: S1 File — (ZIP) [file pone.0237278.s001.zip › supplementary material/low initial uncertainty_low initial prediction error_p11.tif]

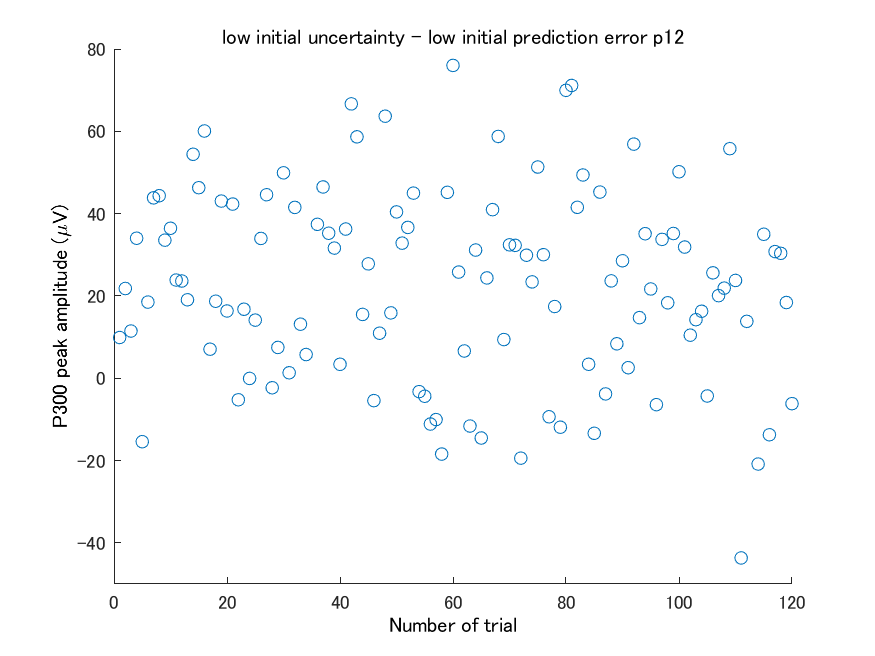

Supplement: S1 File — (ZIP) [file pone.0237278.s001.zip › supplementary material/low initial uncertainty_low initial prediction error_p12.tif]

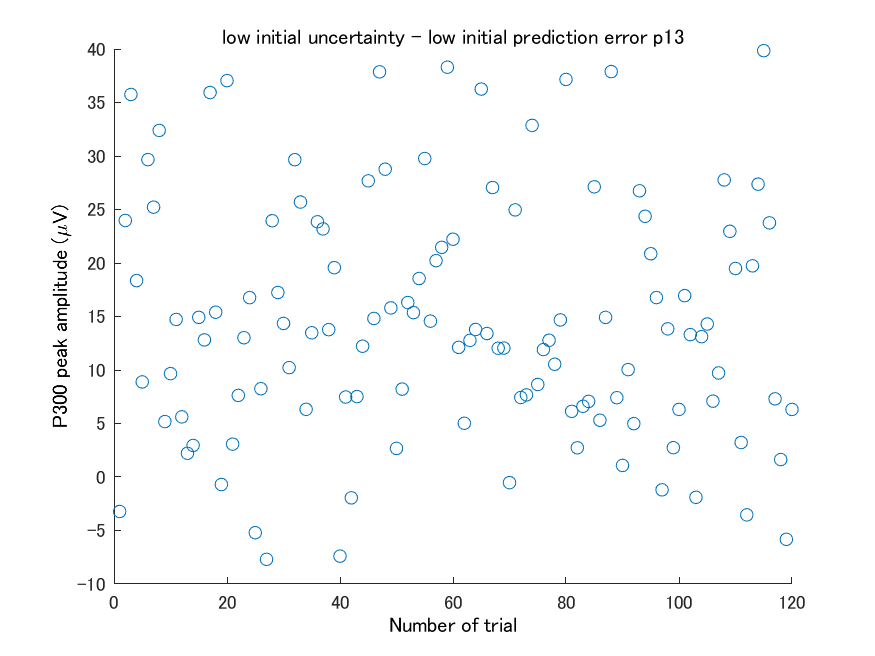

Supplement: S1 File — (ZIP) [file pone.0237278.s001.zip › supplementary material/low initial uncertainty_low initial prediction error_p13.tif]

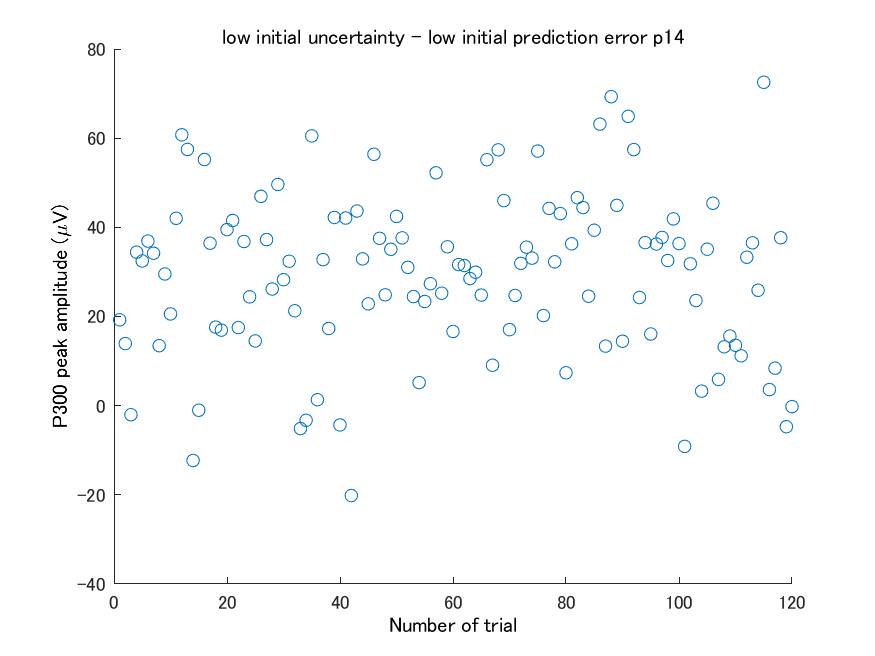

Supplement: S1 File — (ZIP) [file pone.0237278.s001.zip › supplementary material/low initial uncertainty_low initial prediction error_p14.tif]

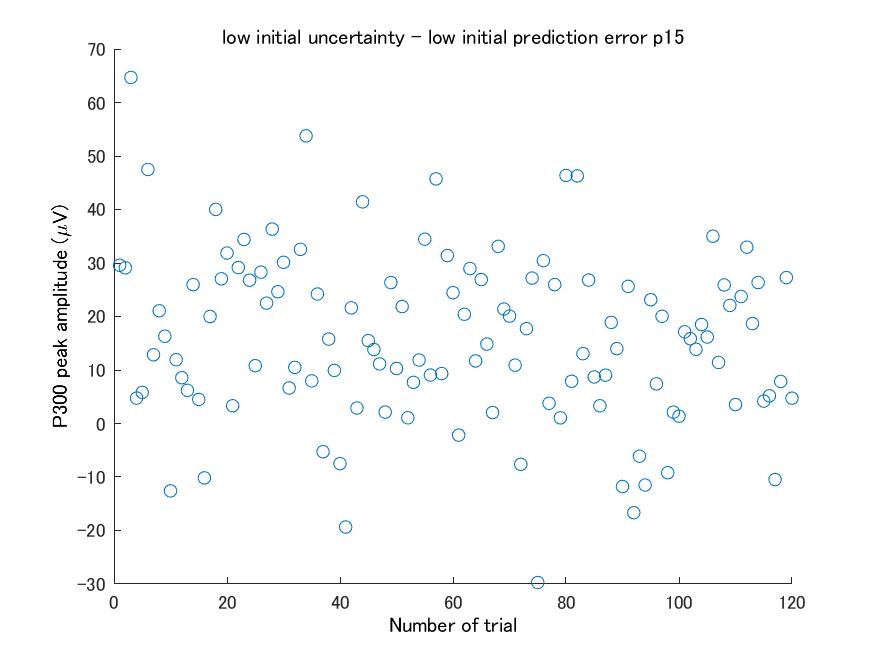

Supplement: S1 File — (ZIP) [file pone.0237278.s001.zip › supplementary material/low initial uncertainty_low initial prediction error_p15.tif]

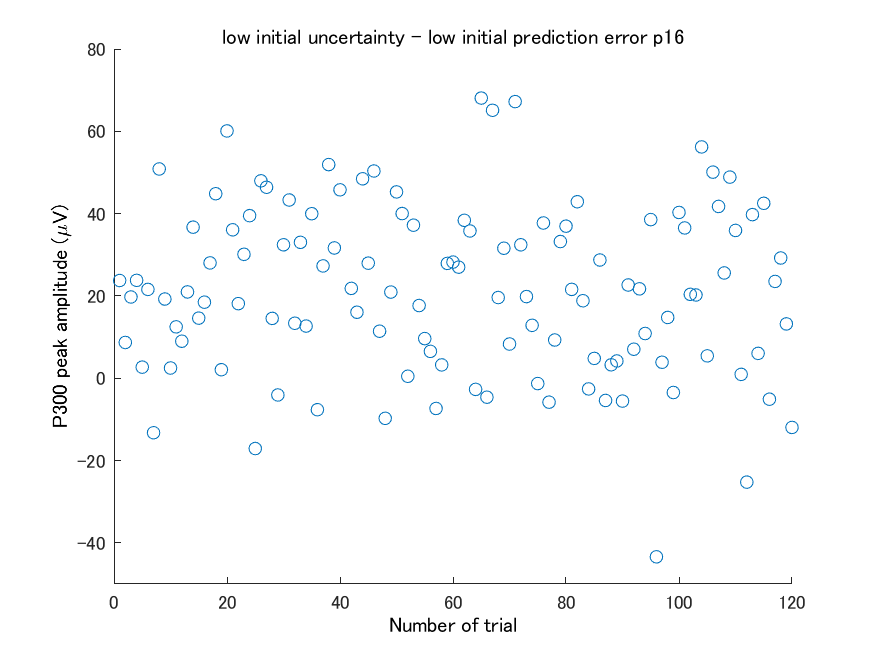

Supplement: S1 File — (ZIP) [file pone.0237278.s001.zip › supplementary material/low initial uncertainty_low initial prediction error_p16.tif]
