# Supplementary material for: How predictability affects habituation to novelty
Source: PLoS One. 2021 Jun 1;16(6):e0237278. doi: 10.1371/journal.pone.0237278 (PMC8168884; doi:10.1371/journal.pone.0237278)
Supplement: S1 Text — (DOCX) [file pone.0237278.s002.docx]

**Supplementary material**

In order to examine the detailed time trends of the P300 amplitude related to habituation, we estimated the P300 amplitude for each trial according to the methods of a previous study [35]. We performed a single-trial regression for each participant using estimates of the P300 amplitude for each condition; however, not all of the regressions were significant (eight significant regressions out of 60 analyses). This may be because only a single P300 amplitude was obtained for each time point for each participant in the experiment, and the background EEG as well as artifacts affected each trial randomly, thereby preventing an accurate regression. Therefore, in this study, the averaging method, which is commonly used in P300 studies, was used to analyze the data with a higher signal-to-noise ratio by reducing the effects of background EEG and artifacts. For reference, a scatter plot for each condition of the time transition of the P300 amplitude estimated in each trial for each participant is shared as supplementary material.
