# Supplementary material for: How predictability affects habituation to novelty
Source: PLoS One. 2021 Jun 1;16(6):e0237278. doi: 10.1371/journal.pone.0237278 (PMC8168884; doi:10.1371/journal.pone.0237278)
Supplement: Attachment — Submitted filename: Response to Editor and Reviewers.docx [file pone.0237278.s003.docx]

Our responses to the editor and reviewers have been uploaded respectively.

To Editor,

(Editor’s comment)

The most pressing concern with the manuscript is the very small sample size—only 8 participants in total, with 7 producing viable data for EEG analysis. Reviewer 1 notes that the sample size is unusually small, even for an ERP study, which raises serious concerns about the study’s capacity to accurately measure the habituation effects of interest. I think the only way to really address this issue is to collect additional data, ideally replicating the original study but with much larger sample.

Response 1:

Following the suggestion of reviewer 1, we conducted an additional experiment to increase the number of participants in the experiment.

For the number of participants in the experiment, we referred to the following paper.

Yano, M., Suwazono, S., Arao, H., Yasunaga, D., & Oishi, H. (2019). Inter-participant variabilities and sample sizes in P300 and P600. International Journal of Psychophysiology, 140, 33-40.

According to Yano et al., International Journal of Psychophysiology (2019), the P300 (P3b) experiment of within-participant factors reported that a significant effect could be reliably detected with a sample size of 7. In this study, we conducted a P300 (P3b) experiment with a within-participant factorial design. However, we further increased the number of participants in the experiment to increase the effect detection power.

(Editor’s comment)

Reviewer 1 also raises concerns about the analysis of the surprise rating data—there is a clear floor effect for the congruent stimuli—which is very likely to be contributing to the key interaction effect described on pg. 11. The reviewer notes that adopting a different method of analysis might be a more appropriate way of analyzing the data, and I encourage you to pursue this. I also wonder whether it might be helpful to examine an “intermediate” level of congruency in an attempt to avoid floor effects on surprise. For example, could you present a modified sound produced by an instrument such that the instrument could not naturally that sound. (Perhaps applying something like a gated reverberation effect could create such an intermediate level of congruency, though feel free to tell me if this is off base.)

Response 2:

In order to avoid floor effects in the subjective evaluation of surprise, we did not consider intermediate levels of congruency, such as the use of modified sounds, because this could change the likelihood function (i.e., the noise that enters the senses), which is a factor that affects information gain.

However, following the suggestion of reviewer 1, we conducted a probit regression analysis, which is a non-parametric analysis, for the subjective evaluation of surprise.

(Editor's comment)

A further concern with the data analysis lies with the binning. I found it a little unusual that the bins were so coarse-grained, given your interest in what appears to be a fairly rapid-onset habituation effect. A better strategy for tackling your key research question would seem to be to opt for more fine-grained aggregation to detect effects that arise early in the learning process. This issue is potentially entangled with the small sample size problem. Reviewer 1 suggests single-trial regression as a potentially more appropriate method of analysis for the kind of data you have.

A final concern I had—also shared by Reviewer 1—is theoretical in nature. You provide a detailed quantitative overview of your Bayesian model, yet you investigate only qualitative predictions (e.g., an interaction effect on P300 amplitude). Finding these effects in the data does not selectively support the information gain hypothesis, and reliance on this kind of reverse inference is not justified. At minimum, there needs to be some consideration of alternative accounts for the data.

Response 3:

Thank you Thank you for your valuable comment.

This study aimed to elucidate how predictability affects habituation to novelty using our mathematical model of arousal that we have already tested, subjective evaluation of surprise, and physiological indices (P300) (Yanagisawa et al., Frontiers in Computational Neuroscience, 2019). Therefore, we are not attempting to propose a new model or perform quantitative reverse inference using P300.

As an additional analysis, we performed a single-trial regression of the P300 amplitude following the method of Mars et al. (2008), but the accuracy of the calculation of P300 amplitude was worse than that of the conventional averaging method of P300 used in our study, so we could not test our hypothesis. With the P300 amplitude using the conventional averaging method, the analysis of 7 participants in the initial draft and 15 participants in the revised draft showed similar results supporting the hypothesis. Therefore, we thought that we could confirm the correspondence between the model predictions and the physiological indices.

In future research, if the goal is to perform inverse inference of the model using the P300 amplitude, it will be necessary to construct an experimental system suitable for single-trial analysis.

Mars, R. B., Debener, S., Gladwin, T. E., Harrison, L. M., Haggard, P., Rothwell, J. C., & Bestmann, S. (2008). Trial-by-trial fluctuations in the event-related electroencephalogram reflect dynamic changes in the degree of surprise. Journal of Neuroscience, 28(47), 12539-12545.

To reviewer 1,

We are grateful to reviewer #1 for the critical comments and useful suggestions that have helped us to improve our paper considerably. As indicated in the response that follow, we have taken the comments and suggestions into account in the revised version of our paper.

1. The empirical section of this manuscript reports a sample of N=8 participants. After exclusion of 1 participant due to excessive eye-blink artefacts, therefore, the final results of this manuscript are derived from a sample of N=7 participants. The effect of this is that the manuscript is severely underpowered to estimate the effect size predicted by its simulations (between initial uncertainty and trial number). This problem is not unique to this manuscript; for instance, Button et al. (Nature Reviews Neuroscience, 2013) discuss the prevalence of underpowered studies in neuroscience more broadly.

There are many consequences of underpowered studies, but the most important one for the present study is that significant results deriving from small samples are likely to significantly overestimate true effect sizes in the population. Notably, this study is underpowered even by the modest standards of ERP research; for instance, Clayson et al. (Psychophysiology, 2019) report the median sample size for ERP studies as 21 participants (3 times that of the sample in this manuscript). Unfortunately, short of replicating results in a larger sample, I believe that this represents a critical shortcoming of the manuscript that precludes interpretation of results.

Response 1:

Thank you for your valuable comment.

According to Yano et al., International Journal of Psychophysiology (2019), the P300 (P3b) experiment of within-participant factors reported that a significant effect could be reliably detected with a sample size of 7. In this study, we conducted a P300 (P3b) experiment with a within-participant factorial design. However, we further increased the number of participants in the experiment to increase the effect detection power. (Clayson et al. (2019) reported on an experiment with a mixed design of between-participant and within-participant factors)

Yano, M., Suwazono, S., Arao, H., Yasunaga, D., & Oishi, H. (2019). Inter-participant variabilities and sample sizes in P300 and P600. International Journal of Psychophysiology, 140, 33-40.

2. The results of the manuscript are presented as a test of a specific computational model of the P300 component of the ERP (in which information gain is operationalised as Kullback-Leibler divergence between posterior and prior beliefs). However, the test of this model presented in the manuscript is qualitative, rather than quantitative:

in both the simulations and the empirical data, there is an interaction between trial number and initial uncertainty. However, such an interaction might also result from other cognitive mechanisms if we assume that their effect is to cause a roughly multiplicative decay in P300 amplitude over time. To give one example, an effect of participant fatigue might also produce the interaction between trial number and initial uncertainty reported in the manuscript.

A far stronger test of the model's predictions would be, rather than discretising trials into 40-trial bins, to run a single-trial regression of P300 amplitudes onto information gain as measured by the manuscript's metric. If this model outperforms one in which P300 amplitude is predicted as a function of trial number (rather than KL divergence), this would constitute stronger evidence for the manuscript's conclusion. This would also be consistent with other recent information-theoretic and Bayesian approaches to analysis of the P300 component (see point 4 below).

Response 2:

Thank you for your valuable comment.

This study aimed to elucidate how predictability affects habituation to novelty using our mathematical model of arousal that we have already tested, subjective evaluation of surprise, and physiological indices (P300) (Yanagisawa et al., Frontiers in Computational Neuroscience, 2019). Therefore, we are not attempting to propose a new model or perform quantitative reverse inference using P300.

However, the reviewer's suggestion was very attractive, so we performed a single-trial regression of the P300 amplitude following the method of Mars et al. (2008). However, the accuracy of estimation of P300 amplitude was worse than that of the conventional averaging method of P300 used in our study, so we could not test our hypothesis. With the P300 amplitude using the conventional averaging method, the analysis of 7 participants in the initial draft and 15 participants in the revised draft showed similar results supporting the hypothesis. Therefore, we thought that we could confirm the correspondence between the model predictions and the physiological indices. However, as pointed out by the reviewer, we cannot deny the effect of fatigue over time, so we stated the following as a limitation of this study.

line 18 in page 15 in “5 Discussion”: “In this study, we experimentally examined habituation associated with repeated presentation of stimuli. Therefore, we cannot deny the possibility that cognitive and physical factors, such as fatigue of the experimental participants over time, may have influenced the interaction between initial uncertainty and initial prediction error. In future experiments, it is necessary to construct an experimental system suitable for single-trial analysis of P300 amplitude, for example, and to examine the time course of habituation in shorter experiments.”

3. An interaction between trial number and initial uncertainty is reported for participants' self-reported surprise ratings. However, I believe that this may be a statistical artefact resulting from the fact that self-report ratings of congruent items are at a floor level (see Figure 5). Indeed, from inspection of the self-report scores in the accompanying supplemental material, it appears that the overwhelming majority of participants reported surprise levels of '1'

(no surprise) for these conditions. In this case it is not surprising that an interaction would occur with initial uncertainty, since surprise can hardly be expected to decrease any further for instruments high in initial certainty. I would also note that in this case, the assumption of the ANOVA (normality of residuals) is violated, and the manuscript might be better served employing a different analytic approach (e.g., probit regression; see Liddell & Kruschke, 2018, Journal of Experimental Social Psychology).

Response 3:

Thank you for your useful suggestion.

Following the suggestion of reviewer 1, we conducted a probit regression analysis, a nonparametric analysis, to test our hypothesis (H1) about the subjective evaluation of surprise. For hypothesis H2, we performed Wilcoxon's signed rank test.

We added the results in line4 page12 in "4 Results".

4. The manuscript's Bayesian model of the amplitude of the P300 is an interesting one, but it does not engage with a body of prior research on Bayesian models of the P300 component of the event-related potential. A Bayesian model of the P300 was first proposed by Kopp (2008; In M. K. Sun (Ed.), Cognitive sciences at the leading edge (pp.

87–96)), and a number of empirical papers testing this and other Bayesian P300 models have been published in the last 5 years (e.g., Kolossa et al., 2013, Frontiers in Human Neuroscience; Kolossa, Kopp & Fingscheidt, 2015, NeuroImage; Bennett et al., 2016, eNeuro; Kopp et al., 2016, Cognitive, Affective, and Behavioural Neuroscience; Bennett et al., 2019, Psychophysiology). The manuscript would be strengthened by engaging with these prior papers that prefigure the ideas proposed here.

Response 4:

Thank you for your useful suggestion.

We cited the paper referred to by the reviewer #1 and added the following sentence in line 21 in page 4.

"Kopp (2007) was the first to study the relationship between Bayesian inference and P300 components (26), and several empirical studies have been conducted since then (27-31). Kolossa et al. showed the correspondence between surprise as the amount of information and P300 (30). They reported that P3a corresponds to KL divergence from Bayesian posterior to prior (termed Bayesian surprise), a change in the probability distribution given a new observation, particularly belief updating about hidden states. On the other hand, P3b is reported to correspond to predictive surprise, meaning surprise about observations under the current probability distribution."

\## Minor points

- Page 4, lines 86-87: "Accordingly, the information gain represents a decrease in uncertainty by experiencing an event". This is not quite correct; KL divergence measures the similarity between two probability distributions. These distributions may be quite dissimilar (i.e., high KL divergence) but still have equal uncertainty (i.e., entropy). For instance, a Beta(10, 1) distribution and a Beta (1,10) distribution have equal entropy but very different KL divergence. See also the equation of information gain with KL divergence on page 3, line 57.

Response 5:

We deleted the part you pointed out (following sentence) as it was incorrect.

"Accordingly, the information gain represents a decrease in uncertainty by experiencing an event."

'- Although the manuscript was well written in general, there were a number of grammatical and spelling lapses throughout. One prominent one is the title of the manuscript ("How predictability affects habituation to novelty?"). If the title is phrased as a question, this should be "How does predictability affect habituation to novelty"; if the title is not phrased as a question then "How predictability affects habituation to novelty" would be grammatical.

Response 6:

We corrected the part you pointed out (Title) as follows to make it grammatically correct.

"How predictability affects habituation to novelty"

The derivations of the Bayesian update rule (Equation 6) and the information gain (Equation 8) are non-trivial, but only the results of these derivations (and not the process by which the equations are

derived) are presented. Speaking personally, this made it rather difficult for me to check the logic of the manuscript's derivation. I would recommend including additional supplementary material detailing these derivations to aid the interested reader.

Response 7:

We added the derivation equation as supplementary material.

To Reviewer 2

We are grateful to reviewer #2 for the critical comments and useful suggestions that have helped us to improve our paper considerably. As indicated in the response that follow, we have taken the comments and suggestions into account in the revised version of our paper.

Appropriate statistical analyses are reported, but for ANOVA I would expect to see some indication of effect size estimates and associated confidence intervals.

Response 1:

Thank you for your useful suggestion.

We included effect size and confidence intervals for the ANOVA we conducted (in line 16 in page 13).

Summary data are available. I would have liked the authors to give access to the stimulus videos also. It is possible to work out what they showed with reference to the methods and table, but the actual stimuli would help.

Response 2:

Thank you for your useful suggestion.

We added the URLs of the stimulus videos used in this study in line 4 in page 10 in "3.1.2 Stimuli".

The manuscript is written in standard English, and is generally intelligible, but I think that the very technical nature of the writing will make it difficult to access by all but a specialist audience. With relatively little effort. I am sure that the authors could make the manuscript accessible to a wider readership by simply explaining key terms and concepts in simpler ways and providing more detail.

The concept of long-term novelty (page 1, line 38) might seem counterintuitive or even oxymoronic to some readers.

Response 3:

As you pointed out, we revised the text as follows to make it more intuitive and easier to understand.

line 20 in page 2 in "1 Introduction": "to maintain an emotional response to novelty."

The authors refer to predictability and uncertainty throughout the introduction of the manuscript, but these terms are first defined at the end of page six, and then in mostly mathematical terms. A clear behavioural/operational definition of predictability and uncertainty when the terms are first used would greatly improve the intelligibility of the manuscript.

Response 4:

As you pointed out, we added a sentence that defines predictability and uncertainty in behavioral and operational terms.

line 20 in page 3 in "1 Introduction": "Uncertainty is an index for the familiarity of an object, the amount of knowledge an individual has, and so on. Prediction error indicates the difference between the prior prediction and the actual sensation."

Clarity of expression could also be improved in places. The opening paragraph of the introduction, for example, states (lines 36-37) “Therefore, if one is experiencing unpleasant novel events, one should get used to them earlier; if one is experiencing pleasant novel events, one should be as unaccustomed as possible.” I assume that the authors mean that it would be desirable/advantageous for the individual to get used to novel unpleasant events as soon as possible to reduce their impact, but the way this sentence is written, it could be interpreted as meaning that novel unpleasant events lead to rapid habituation.

Response 5:

In order to avoid misunderstandings due to ambiguity, we corrected the parts you pointed out.

line 18 in page 2 in "1 Introduction": "Therefore, if one is experiencing unpleasant novel events, one should get used to them as soon as possible"

In the procedure (3.1.3), the authors refer to eight videos, each shown 60 times, but then in the analysis section (3.1.5) mention four stimulus types and 120 exposures. It took me longer than it should have to work out that there were two videos of each stimulus type – a statement to this effect in the procedure would have helped.

Response 6:

As you pointed out, it is difficult to understand, so we added the following sentence.

line 7 in page 11 in "3.1.3 Procedure": "Each of the four stimulus types (i.e., AX, AY, BX, and BY) contains two videos (Table 1)."
